# Comparative effectiveness of exercise and self-management interventions delivered through different modes of digital support on pain and patient-reported physical function in people with knee osteoarthritis: a systematic review and network meta-analysis of randomised controlled trials

**DOI:** 10.3389/fmed.2026.1956102

**Published:** 2026-09-30

**Authors:** Hao Luo, Guoqing Zhang, Lingyun Long, Wenjia Chen, Jiayi Yao, Yong Liu

**Affiliations:** 1 Department of Physical Education, Chongqing Electric Power College, Chongqing, China; 2 School of Physical Education, China University of Mining and Technology, Xuzhou, China

**Keywords:** digital health, exercise, knee osteoarthritis, network meta-analysis, self-management, telerehabilitation

## Abstract

**Objective:**

Previous reviews have commonly pooled heterogeneous digital technologies, leaving the relative effects of different delivery approaches, feedback mechanisms, and levels of professional involvement unclear. This study compared seven exercise and self-management strategies for WOMAC pain, WOMAC physical function, and retention at the end of the intervention in people with knee osteoarthritis.

**Methods:**

Seven databases were searched through July 2026 for randomised controlled trials involving adults with knee osteoarthritis. Interventions were classified into seven nodes according to exercise delivery, feedback mechanisms, and professional involvement. Frequentist random-effects network meta-analyses were conducted, with WOMAC outcomes expressed as Hedges' g and retention as risk ratios. Risk of bias and certainty of evidence were assessed using RoB 2 and GRADE.

**Results:**

Twenty-one RCTs involving 2,250 participants were included. The pain, physical function, and retention networks comprised 8, 12, and 20 trials, respectively. For physical function, D1 vs. C1 (g = 0.47, 95% CI 0.31 to 0.64) and D2 vs. C2 (g = 0.47, 95% CI 0.22 to 0.72) showed improvements, with moderate-certainty evidence. The estimate for D3 vs. C3 was imprecise (g = 0.48, 95% CI −0.13 to 1.10; low-certainty evidence). All three pain comparisons favoured the digitally supported nodes (g = 0.15, 0.21, and 0.50), but all 95% CIs included the null value, with low-certainty evidence. Retention differences for D2 vs. C2 and D3 vs. C3 were likely to be small, whereas local inconsistency was identified for D1 vs. C1.

**Conclusion:**

Comparison-specific network estimates favoured D1 over C1 and D2 over C2 for patient-reported physical function, but these contrasts used different comparators and do not establish superiority of digital delivery itself or the independent value of professional support. The incremental value of synchronous or hybrid professional support over face-to-face supervised exercise remains uncertain. Incremental effects on WOMAC pain remain unclear, and retention estimates were generally close to the null, with local inconsistency for D1 vs. C1. Current evidence does not support prioritising synchronous or hybrid professional support solely on the basis of rankings or assuming a linear relationship between digital-support intensity and treatment effects.

**Systematic Review Registration:**

www.crd.york.ac.uk/prospero, identifier CRD420261467021.

## 1 Introduction

Knee osteoarthritis is a common chronic musculoskeletal disorder characterised primarily by pain, stiffness, and restricted mobility, which may further impair activities of daily living and health-related quality of life (1–3). With population ageing and the increasing prevalence of risk factors such as obesity, its long-term disease burden and demand for healthcare services are expected to continue rising. Current guidelines consistently identify patient education, self-management, and structured exercise as core components of non-surgical management for knee osteoarthritis. Exercise programmes should include an appropriate dose and progression and should be tailored to patients' functional status, personal preferences, and available services (4–6). However, conventional face-to-face exercise management often depends on fixed locations and sustained professional input. Patients may also face barriers related to travel distance, scheduling, treatment costs, and limited access to professional services, restricting both access to exercise prescriptions and their sustained implementation (7–9).

Digital technologies provide more flexible approaches to delivering exercise and self-management interventions for knee osteoarthritis. Existing interventions may use mobile applications, websites, videos, text messaging, telephone calls, wearable devices, or sensors to provide exercise instruction, automated reminders, self-monitoring, progress tracking, and remote feedback, potentially reducing temporal and geographical barriers and extending the reach of rehabilitation services (10–12). However, “digital interventions” do not constitute a single homogeneous treatment category. Substantial differences exist between programmes in whether they include structured exercise, adapt prescriptions according to patient performance, provide ongoing individualised feedback, and involve non-real-time asynchronous support or real-time synchronous professional guidance (13–15). The same technological platform may deliver different support mechanisms, whereas different technologies may perform similar clinical functions (16–18). Therefore, the clinical relevance of digitally supported exercise may depend less on the specific device or product brand than on how structured exercise is delivered, supported by feedback, and integrated with professional involvement.

Previous systematic reviews have commonly pooled interventions delivered through mobile applications, websites, telephone calls, text messaging, videos, and wearable devices into broad categories of telerehabilitation or digital health interventions, primarily comparing them with usual care, health education, or conventional rehabilitation (19–21). A 2024 meta-analysis suggested that remotely supported exercise may improve pain and physical function in people with knee osteoarthritis, although the certainty of evidence was low and heterogeneity was present across digital technologies and comparator types. Other reviews have reached less consistent conclusions regarding improvements in physical function (10, 14, 15). Existing research has also rarely distinguished automated self-directed exercise, asynchronous professional feedback, and synchronous professional guidance as separate mechanisms, leaving uncertainty as to whether greater human involvement provides clear incremental value (7, 13, 22). In addition, study completion, withdrawal, and actual exercise performance are often reported together, making it difficult to distinguish the effects of digital interventions on retention from their effects on exercise adherence. Conventional pairwise meta-analysis mainly synthesises available direct comparisons and is therefore limited in its ability to compare multiple digital and non-digital strategies simultaneously or to determine whether observed benefits arise from structured exercise itself, the digital mode of delivery, or additional professional support.

Network meta-analysis can integrate direct and indirect evidence, provided that the assumption of clinical exchangeability is met, and offers a unified framework for comparing multiple exercise-support strategies (23–25). Accordingly, rather than classifying interventions according to mobile applications, websites, videos, or device brands, we constructed seven clinically interpretable nodes based on the delivery of structured exercise, feedback mechanisms, and the degree of professional involvement. WOMAC pain and WOMAC physical function were designated as co-primary outcomes, and retention at the end of the intervention was considered the key secondary outcome. In addition to using usual care as the network reference, we prespecified three mechanism-matched comparisons: self-directed digital exercise vs. minimal-intensity digital education, digitally delivered exercise with asynchronous professional support vs. non-digital self-directed exercise, and synchronous or hybrid professional support vs. face-to-face professionally supervised exercise. This study aimed to estimate the relative effects of the seven nodes, evaluate the incremental value of different modes of digital support over mechanism-matched comparators, and determine whether the available evidence supports the clinical prioritisation of particular digital-support approaches by considering treatment rankings, risk of bias, inconsistency, sensitivity analyses, and GRADE certainty of evidence.

## 2 Materials and methods

The study protocol was registered with PROSPERO (CRD420261467021), with the study objectives, eligibility criteria, interventions and comparators, and planned outcomes specified in advance. This systematic review and network meta-analysis was conducted in accordance with the registered protocol and reported following the PRISMA 2020 statement and the PRISMA extension for network meta-analyses. The literature search was reported in accordance with the PRISMA-S guidelines (26–28).

### 2.1 Search strategy

Seven databases—PubMed, Web of Science, Embase, the Cochrane Library, Scopus, CINAHL, and Wiley Online Library—were systematically searched from inception to 10 July 2026. The search strategy was initially developed for PubMed and subsequently adapted according to the controlled vocabulary and search syntax of each database. Subject headings were combined with free-text terms in titles and abstracts, covering four core concepts: knee osteoarthritis, digital health or telerehabilitation, exercise and self-management interventions, and randomised controlled trials. The principal search terms included “knee osteoarthritis”, “digital health”, “telerehabilitation”, “mobile application”, “web-based”, “exercise”, “rehabilitation”, “self-management”, and “randomized controlled trial”, combined using Boolean operators. The reference lists of included studies and relevant systematic reviews were also screened manually. The complete search strategies, search dates, and numbers of records retrieved are provided in Supplementary Table S1 and are consistent with the PRISMA flow diagram. No separate systematic search of clinical trial registries (e.g., ClinicalTrials.gov) or dedicated grey-literature databases was undertaken.

### 2.2 Eligibility criteria

Eligibility criteria were defined according to the PICOS framework. The population comprised adults with knee osteoarthritis diagnosed using clinical or radiographic criteria, a physician diagnosis, or recognised guideline criteria, with no restrictions on age, symptom duration, or Kellgren–Lawrence grade. Studies involving rehabilitation before or after joint replacement, acute knee injury, inflammatory arthritis, predominantly postoperative pain, or mixed populations from which knee osteoarthritis data could not be extracted separately were excluded (4, 29, 30). Eligible interventions were required to include planned, structured, and repeatedly performed exercise. Digital technology had to play a substantive role in the delivery, guidance, monitoring, or feedback of the exercise prescription, in behavioural support, or in facilitating contact with healthcare professionals. Interventions could additionally include education, pain management, goal setting, and other self-management components. Eligible comparators included usual care, a waiting-list control, minimal-intensity digital education, non-digital self-directed exercise, face-to-face professionally supervised exercise, or another digitally supported strategy meeting the predefined node criteria.

Each randomised arm was classified into one of seven nodes according to the mode of exercise delivery, feedback mechanism, and degree of professional involvement. C0 comprised usual care, a waiting-list control, or no structured exercise prescribed by the study. C1 comprised minimal-intensity digital education without a structured digital exercise prescription. C2 comprised non-digital self-directed exercise without ongoing professional supervision. C3 comprised face-to-face professionally supervised exercise. D1 comprised self-directed digital exercise without ongoing individualised human feedback. D2 comprised digitally delivered exercise with asynchronous support involving non-real-time professional feedback. D3 comprised digitally delivered exercise with synchronous or hybrid professional support through real-time video guidance or planned face-to-face contact. Nodes were defined by their support mechanisms rather than by the technological platform used. The co-primary outcomes were WOMAC pain and WOMAC physical function, and the key secondary outcome was retention at the end of the intervention. Exercise adherence was not included as an outcome in this study. Only randomised controlled trials were included, comprising parallel-group, multi-arm, cluster, pilot, and feasibility RCTs. Quasi-randomised, non-randomised, single-arm studies, case series, protocol papers, and studies from which an eligible effect estimate could not be derived were excluded. Node assignment followed a prespecified hierarchical algorithm: first, whether a study-prescribed structured exercise programme was present; second, whether exercise was delivered digitally or non-digitally; third, whether ongoing individualised human clinical support was absent or present; and fourth, whether professional support was asynchronous or synchronous/hybrid. Automated reminders, self-monitoring, algorithmic progression, or sensor-generated feedback without planned ongoing human review remained D1. Non-real-time clinician review, messaging, telephone contact, or remote prescription adjustment was classified as D2. Real-time video supervision or planned repeated face-to-face professional contact integrated into the digital programme was classified as D3. One-off orientation, technical support, or generic education common to both arms did not determine node membership. Education, pain management, goal setting, and behavioural support were extracted as co-interventions and were considered in the transitivity assessment; they did not override node assignment unless they altered the exercise-delivery, feedback, or professional-contact mechanism. Accordingly, the nodes represent multicomponent delivery strategies rather than isolated effects of digital technology.

### 2.3 Study selection and data collection

After duplicate records had been removed, two reviewers independently screened titles and abstracts and assessed potentially eligible full-text articles against the prespecified eligibility criteria. Disagreements were resolved through discussion, with arbitration by a third reviewer when necessary. Reasons for exclusion at the full-text stage were recorded for each study. Multiple reports arising from the same randomisation process were consolidated as a single independent trial and assigned a unique Trial_ID to avoid duplicate weighting.

Using a prespecified data extraction form, two reviewers independently extracted information on authorship, year of publication, country, study design, sample size, demographic characteristics, diagnostic criteria, and disease severity. Intervention characteristics included exercise type, frequency, session duration, intervention duration, digital platform, feedback modality, mode of professional contact, and comparator characteristics. Outcome data included the WOMAC version, scoring range, assessment time point, mean, standard deviation, outcome-specific sample size, number of participants retained at the end of the intervention, and number randomised. Each randomised arm was mapped to a prespecified node according to the intervention described in the original report, and the rationale for classification and the resolution of any disagreements were documented.

When a study reported multiple eligible assessment time points, the end-of-intervention assessment designated by the study authors was prioritised. When this was not explicitly specified, the assessment closest to the end of the intervention was selected; if two assessments were equally close, the earlier first post-intervention assessment was used. Time points were not selected according to the direction or statistical significance of the effect. For continuous outcomes, directly usable baseline-adjusted between-group estimates were prioritised when available; otherwise arm-level change scores were used, with follow-up endpoint data retained as a prespecified fallback. Missing standard deviations were sought from the main report and Supplementary Materials and, when necessary, reconstructed from convertible statistics. When a change-score standard deviation was unavailable, it was derived from baseline and follow-up standard deviations using a study-specific correlation when recoverable and the prespecified r = 0.50 otherwise. Standardised effects were calculated as Hedges g with the small-sample correction. In the frozen WOMAC pain and physical-function datasets used for the primary analyses, all contributing arm-level inputs were change scores, so endpoint data were not required. Detailed equations, variance assumptions, data-conversion rules, study-level extracted inputs, and derived direct effects are provided in the Supplementary Methods and Supplementary Table S10. For Barton et al. (63), the intervention lasted 6–8 weeks and the trial scheduled its first follow-up at 3 months from intervention commencement; no assessment was scheduled exactly at programme completion. The 3-month assessment, approximately 4–6 weeks after programme completion, was therefore used as the closest available assessment for retention.

### 2.4 Risk of bias and certainty of evidence

Two reviewers independently assessed the risk of bias for the effect of assignment to intervention in each trial at the target outcome and assessment time point using the Cochrane RoB 2 tool (31). Five domains were evaluated: bias arising from the randomisation process, bias due to deviations from intended interventions, bias due to missing outcome data, bias in measurement of the outcome, and bias in selection of the reported result. Each domain and the overall risk of bias were judged as “low risk”, “some concerns”, or “high risk”. Disagreements were resolved through discussion, with arbitration by a third reviewer when necessary.

The certainty of evidence from the network meta-analysis was assessed using the GRADE approach (32, 33). The unit of assessment was the outcome-specific network comparison. Evidence from randomised controlled trials initially started at high certainty and was downgraded according to study limitations, inconsistency, indirectness, imprecision, and publication bias (34, 35). For each key network estimate, the assessment explicitly considered the amount and risk of bias of the direct evidence, the availability and precision of indirect evidence, node-splitting results when direct and indirect evidence coexisted, the clinical plausibility of transitivity, and the width and location of the network confidence interval. Nominal network connectivity was not treated as increasing certainty when indirect evidence was absent or extremely imprecise. Particular emphasis was placed on comparisons of each node with C0 and on D1 vs. C1, D2 vs. C2, and D3 vs. C3. For WOMAC outcomes, Hedges g = 0 was treated as the null value. The conventional |g| approximately 0.20 benchmark was retained only as a descriptive reference for standardised effect magnitude and was not treated as a WOMAC-specific minimal clinically important difference or as the sole criterion for GRADE imprecision or clinical importance. Imprecision was judged from the width of the 95% CI, inclusion of the null, and whether the range of plausible effects would lead to materially different interpretations. For retention, RR = 1 was the null value; the prespecified 0.83 and 1.20 values were treated as contextual boundaries rather than as thresholds for adherence or acceptability. Table 4 reports the direct-evidence volume and the status of indirect evidence for each key comparison rather than repeating the size of the entire outcome network. A single outcome-specific WOMAC MCID was not imposed *post hoc* because the included WOMAC formats and within-study standard deviations differed, and conversion of a point-based MCID to the standardised scale would therefore be study dependent. This limitation is reflected in the more cautious interpretation of clinical importance.

### 2.5 Statistical analysis

Because the included studies used different WOMAC versions, response formats, scoring ranges, and change-score dispersions, pain and physical function were analysed using standardised mean differences with Hedges' small-sample correction and expressed as Hedges g with 95% confidence intervals. Simple linear rescaling to a common 0–100 metric would harmonise the numerical range but would not address between-study differences in score dispersion and measurement properties; the standardised effect was therefore prespecified to synthesise the same WOMAC constructs across heterogeneous formats. This choice reduces direct clinical interpretability, and Hedges g was consequently interpreted as a standardised relative effect rather than as a WOMAC-point minimal clinically important difference. After harmonisation of effect direction, g > 0 indicated a more favourable effect for the first-listed node. Retention was defined as the proportion of randomised participants who completed the prespecified end-of-intervention (or closest available post-intervention) assessment, using the number randomised as the denominator. It was prioritised because randomised and assessment-completion counts were consistently extractable across nearly all included trials, providing a pragmatic trial-completion outcome. Retention was not interpreted as exercise adherence, sustained exercise engagement, or intervention acceptability. Effects were expressed as risk ratios with 95% confidence intervals, with RR > 1 indicating higher retention for the first-listed node.

Separate frequentist contrast-based random-effects network meta-analyses were conducted for WOMAC pain, WOMAC physical function, and retention, with a common between-study heterogeneity variance assumed within each network. All eligible randomised arms from multi-arm trials were retained, and correlations arising from shared comparators were accounted for within the model. C0 was used as the reference node for the overall network, while interpretation focused particularly on the three mechanism-matched comparisons of D1 vs. C1, D2 vs. C2, and D3 vs. C3. Because these comparisons used different comparator nodes, their effect estimates were not interpreted as representing a linear dose–response relationship with increasing intensity of digital support.

Network heterogeneity was quantified using *τ*². Transitivity was assessed descriptively by comparing potential effect modifiers across direct comparisons, including age, sex, body mass index, baseline symptom severity, radiographic severity, exercise type and dose, intervention duration, professional contact, co-interventions, and comparator intensity. Because most direct comparisons were informed by few trials and several covariates were incompletely reported, formal statistical equality tests or network meta-regression were not considered reliable. The observed distributions are summarised in Supplementary Tables S9 and S11. There was broad overlap in age and radiographic severity where reported, but important residual imbalances remained, including women-only trials in selected comparisons, intervention durations ranging from 4 to 24 weeks, differences in exercise dose and progression, variation in professional contact and behavioural support, and marked differences in comparator intensity from usual care to face-to-face supervised exercise. Transitivity was therefore treated as an assumption with residual uncertainty rather than as empirically established. A global inconsistency model was used to assess disagreement between direct and indirect evidence, and node-splitting analyses were conducted for comparisons informed by both direct and indirect evidence. Findings were interpreted by considering effect direction and magnitude, confidence intervals, network sparsity, and clinical exchangeability rather than treating *P* > 0.05 as evidence of complete consistency.

SUCRA values, mean ranks, and cumulative ranking probabilities were calculated for the two WOMAC outcomes as exploratory summaries. Cumulative rank-probability curves were used to display ranking uncertainty, and SUCRA, PrBest, or mean rank were not used to infer clinical superiority or to generate treatment recommendations. Rankings for sparsely represented nodes, particularly D3, were considered unstable. Leave-one-out influence analyses were conducted by sequentially removing each independent trial and refitting the network. Additional *post hoc* sensitivity analyses excluded trials judged at high overall risk of bias and examined alternative plausible node assignments recorded as classification uncertainties in the analysis log. As a conservative structural sensitivity analysis, Kotnik et al. (38) was alternatively reclassified from D2 to D3 because the intervention allowed optional synchronous video consultation, and for retention the Nambi et al. (70) control was alternatively reclassified from C2 to C3 because the report contained conflicting statements regarding weekly supervision. No separate WOMAC data-type sensitivity analysis was required because the frozen WOMAC networks used change-score inputs throughout. *Post hoc* cut-points for intervention duration or comparator intensity were not imposed because they were not prespecified and would further fragment the sparse WOMAC networks; these characteristics were instead examined in the transitivity assessment. Because the WOMAC pain network included only eight RCTs, no regression-based test for small-study effects was performed; a comparison-adjusted funnel plot was generated *post hoc* for exploratory presentation only. Comparison-adjusted funnel plots and exploratory regression diagnostics were conducted for WOMAC physical function and retention. The absence of a clear signal was not interpreted as evidence that publication bias was absent.

The primary network analyses, league tables, treatment rankings, and inconsistency assessments were performed in Stata 18. Supplementary leave-one-out analyses and funnel plots were generated from the same frozen dataset using reproducible scripts. All tests were two-sided, with *P* < 0.05 used as a statistical reference threshold; however, statistical significance was not used as the sole criterion for judging clinical importance, network consistency, or certainty of evidence.

## 3 Results

### 3.1 Literature search and study selection

A total of 4,489 records were identified through searches of the seven electronic databases. After removal of 3,268 duplicate records, 1,221 records underwent title and abstract screening. Of these, 1,103 were excluded because they did not meet the eligibility criteria, leaving 118 articles for full-text assessment.

All 118 full-text articles were successfully retrieved. Following full-text assessment, 97 articles were excluded: 31 were not randomised controlled trials, 19 included ineligible populations, 24 evaluated ineligible interventions, and 23 did not report the outcomes of interest. Ultimately, 21 study reports corresponding to 21 independent randomised controlled trials were included, comprising 2,250 participants with knee osteoarthritis. The study selection process is presented in Figure 1.

**Figure 1:**
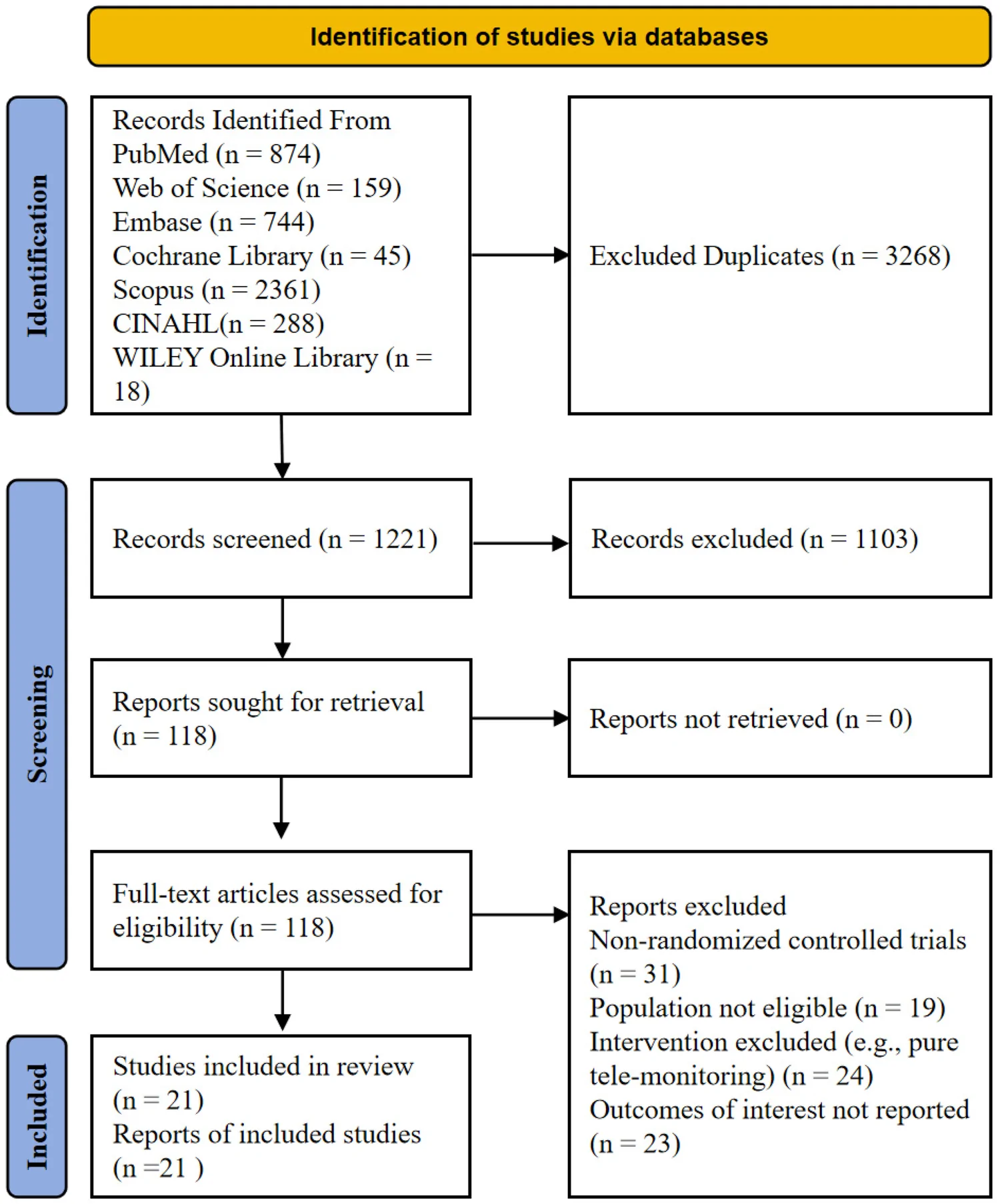
PRISMA 2020 flow diagram of study identification, screening, eligibility assessment, and inclusion.

### 3.2 Characteristics of the included studies

The 21 included RCTs were published between 2018 and 2026 and were conducted across 13 countries or regions. Five studies were conducted in Australia, while two studies each were conducted in the United States, Saudi Arabia, Germany, and Türkiye. A total of 2,250 participants with knee osteoarthritis were randomised. Individual study sample sizes ranged from 29 to 350 participants, and mean participant ages across study groups ranged from approximately 51.5 to 71.4 years. Most trials included both men and women, whereas two enrolled women only. Knee osteoarthritis was primarily confirmed using the American College of Rheumatology or NICE clinical criteria, physician diagnosis, or radiographic evidence. Among studies reporting radiographic severity, most included participants with Kellgren–Lawrence grades I–III.

Most studies were conducted in home-based or community telerehabilitation settings, although some included face-to-face exercise comparators delivered in outpatient or clinical settings. Intervention duration ranged from 4 to 24 weeks, with most interventions lasting 6–12 weeks. Exercise frequency ranged from two or three sessions per week to daily exercise.

Randomised arms were classified into seven nodes according to the digital-support mechanism and mode of professional involvement. C0–C3 represented usual care or waiting-list control, minimal-intensity digital education, non-digital self-directed exercise, and face-to-face professionally supervised exercise, respectively. D1 represented self-directed digital exercise, D2 represented digitally delivered exercise with asynchronous professional support, and D3 represented digitally delivered exercise with synchronous or hybrid professional support. Digital platforms included websites, mobile applications, videos, text messaging, sensors, wearable devices, and virtual reality; however, nodes were defined according to the mode of exercise delivery and feedback rather than the technology brand.

The WOMAC pain network included 8 RCTs, 943 participants, and 17 randomised arms. The WOMAC physical function network included 12 RCTs, 1,454 participants, and 25 randomised arms. The retention network included 20 RCTs, 2,189 participants, and 41 randomised arms. Allen 2018 was a three-arm trial, and the correlation arising from its shared comparator was accounted for within the network model. Study characteristics are presented in Table 1, and detailed node definitions and arm-level study mappings are provided in Supplementary Table S2. Although all seven nodes were mathematically connected, this should not be interpreted as an adequately informative evidence network: most direct comparisons were supported by a single trial, D3 was represented by one 21-participant intervention arm, and some indirect paths were subsequently shown to have extremely large standard errors.

**Table 1:** Characteristics of the included randomised controlled trials.

Study	Participants	Digital intervention (node)	Comparator (node)	Programme duration and assessment	NMA outcome(s)
Aily et al. (2023) (51)	*N* = 100 (50/50); age 53.0 (9.0)/55.0 (8.0)	D2: Pre-recorded home circuit training plus scheduled PT telephone support	C3: Identical circuit training supervised face to face by a PT	14 wk; assessed at 14 and 26 wk	F; R
Alasfour et al. (2022) (61)	*N* = 40 women (20/20); age 53.65 (3.96)/55.15 (4.64)	D2: App-guided strengthening with remote therapist monitoring	C2: Identical paper-based home exercise	6 wk; assessed at 3 and 6 wk	F; R
Allen et al. (2018) (62)	*N* = 350 (142/140/68); age 65.3 (11.5)/65.7 (10.3)/64.3 (12.2)	D1: Automated, individually tailored web-based exercise (IBET)	C3: In-person individualised PT; C0: waiting list/usual care	4 mo; assessed at 4 and 12 mo	P; F; R
Barton et al. (2026) (63)	*N* = 44 (22/22); age 62 (8)/64 (10)	D3: Live group GLA:D exercise and education by videoconference	C3: Identical group GLA:D programme delivered face to face	6–8 wk; first follow-up at 3 mo from intervention commencement (≈4–6 wk after programme completion; used for retention); later follow-ups at 12 and 24 mo	R
Bennell et al. (2022) (64)	*N* = 212 (107/105); age 62.8 (8.2)/61.8 (7.2)	D1: Self-directed online yoga with automated reminders	C1: Online knee osteoarthritis education	12 wk; assessed at 12 and 24 wk	P; F; R
Bennell et al. (2020) (65)	*N* = 110 (56/54); age 61.7 (6.7)/62.9 (6.8)	D1: Unsupervised home exercise plus tailored automated text messages	C2: Identical home exercise without text-message support	24 wk; assessed at 24 wk	R
Cai et al. (2026) (66)	*N* = 35 (16/19); age 59.64 (6.70)/66.33 (8.53)	D3: Wearable- and app-supported exercise with live video guidance and scheduled in-person review	C3: In-person PT with paper-based home exercise	12 wk; assessed at 12, 24, and 36 wk	R
Dieter et al. (2024) (45)	*N* = 61 (15/15/31); age 61.5 (7.5) for digital arms/64.2 (9.3) for usual care	D1: Sensor-guided app exercise (app-only arm)	C0: Waiting list/usual care. The app-plus-brace arm was excluded from the primary network because the brace constituted an additional active co-intervention.	12 wk; assessed at 12 wk	—
Dieter et al. (2025) (67)	*N* = 195 (98/97); age 61.6 (8.0)/62.1 (7.5)	D1: Self-directed sensor-guided app exercise with automated real-time feedback	C0: Usual care without a study-prescribed exercise programme	12 wk; assessed at 3 mo	R
Gohir et al. (2021) (68)	*N* = 146 (67/79); age 65.2 (9.7)/68.0 (8.6)	D2: Adaptive app-based exercise with asynchronous therapist chat or telephone support	C0: Usual self-management and routine care	6 wk; assessed at 6 wk	P; F; R
Im et al. (2025) (69)	*N* = 29 (19/10); age 69.9 (6.8)/71.4 (6.4)	D2: App-based exercise with weekly clinician data review and dose adjustment	C2: Identical paper-based self-directed exercise	6 wk; assessed at 6 wk	P; F; R
Kotnik et al. (2026) (38)	*N* = 151 randomised (66/85); 118 analysed at 12 wk (57/61); age 50–79; mean NR	D2: Web-based exercise with asynchronous contact and optional video consultation	C2: Paper-based exercise with one mid-programme telephone contact	12 wk; assessed at 12 wk	P; F; R
Lo et al. (2024) (36)	*N* = 30 (15/15); median age 63 (IQR 60–67)/64 (IQR 62–65)	D1: Self-directed immersive virtual-reality strengthening with automated feedback	C2: Identical paper-based strengthening exercise	12 wk; assessed at 6 and 12 wk	P; F; R
Nambi et al. (2026) (70)	*N* = 56 women (28/28); age 56.1 (4.8)/57.5 (4.6)	D3: App-based exercise with real-time video supervision and progression	C2: Paper-based self-directed exercise	8 wk; assessed at 2 and 6 mo	R
Nelligan et al. (2021) (71)	*N* = 206 (103/103); age 60.3 (8.2)/59.0 (8.5)	D1: Web-based strengthening plus automated behaviour-change text messages	C1: Web-based knee osteoarthritis education	24 wk; assessed at 24 wk	F; R
Plavoukou et al. (2025) (40)	*N* = 42 (21/21); age reporting internally inconsistent	D3: Live sensor-augmented telerehabilitation supervised by a PT	C3: Identical exercise supervised face to face by a PT	8 wk; assessed at 8 wk and 3 mo after intervention	P; F; R
Tedeschi et al. (2024) (43)	*N* = 55 (29/26); age 58.6 (17.4)/63.6 (11.0)	D3: Real-time video-supervised therapeutic exercise	C3: Identical exercise supervised in an outpatient clinic	4 wk; assessed at 4 wk and 3 mo	R
Tore et al.(2023) (47)	*N* = 50 (25/25); age 55.87 (7.24)/55.79 (6.76)	D3: Live video-supervised exercise with immediate movement correction	C2: Identical paper-based self-directed home exercise	8 wk; assessed at 8 wk	R
Tümtürk et al. (2024) (37)	*N* = 57 (29/28); age 53.59 (7.12)/51.50 (7.03)	D2: WhatsApp-delivered exercise with weekly telephone support and adjustment	C2: Identical paper-based self-directed exercise	8 wk; assessed at 8 wk	P; F; R
White et al. (2025) (41)	*N* = 103 (51/52); age 60.7 (10.1)/61.2 (8.0)	D3: Five individual video PT sessions plus Fitbit-supported home exercise	C1: Self-directed web-based OA resources without an individualised structured exercise prescription or ongoing PT contact	12 wk; assessed at 12 and 24 wk	R
Zhu et al. (2026) (72)	*N* = 178 (89/89); age 62.1 (7.3)/61.0 (8.7)	D1: Self-directed online Tai Chi plus automated behavioural support	C1: Online knee osteoarthritis education	12 wk; assessed at 12 wk	F; R

Detailed component coding for exercise type, dose/progression, education, behavioural support, therapist contact, automated feedback, and other co-interventions is provided in Supplementary Table S9.

P, WOMAC pain; F, WOMAC physical function; R, retention; PT, physiotherapist; NR, not reported.

#### 3.2.1 Transitivity and intervention-component assessment

Review of potential effect modifiers showed overlap across many comparisons but also clinically relevant imbalances. Mean or median ages across contributing groups ranged from approximately 51.5 to 71.4 years, and women-only populations occurred in selected comparisons. Radiographic severity was commonly within Kellgren–Lawrence grades I–III when reported, but reporting was incomplete. Intervention duration ranged from 4 to 24 weeks; exercise content and progression varied from strengthening and range-of-motion programmes to yoga, Tai Chi, virtual-reality strengthening, and sensor-guided neuromuscular exercise. Comparator intensity also differed substantially across C0, C1, C2, and C3. Education, pain-management content, behavioural support, automated feedback, and professional contact were not uniformly distributed. These differences mean that mathematical network connectivity should not be equated with complete clinical exchangeability. Detailed arm-level intervention components and a comparison-level transitivity summary are provided in Supplementary Tables S9 and S11.

### 3.3 Risk-of-bias assessment

The risk of bias of the 21 included RCTs was assessed using the Cochrane RoB 2 tool (31). Overall, 16 studies were judged to raise “some concerns”, five were judged to be at “high risk”, and none were judged to be at low risk of bias. The principal concerns related to outcome measurement and missing outcome data. In particular, patient-reported WOMAC outcomes in open-label exercise interventions may have been influenced by participants' awareness of treatment allocation. Study-level and domain-specific judgements are presented in Figures 2A,B. Because no included trial was judged at overall low risk of bias, a low-risk-only analysis was not possible. A *post hoc* sensitivity analysis excluding the five trials at overall high risk of bias was therefore conducted and is reported in Section 3.8 and Supplementary Table S12.

**Figure 2:**
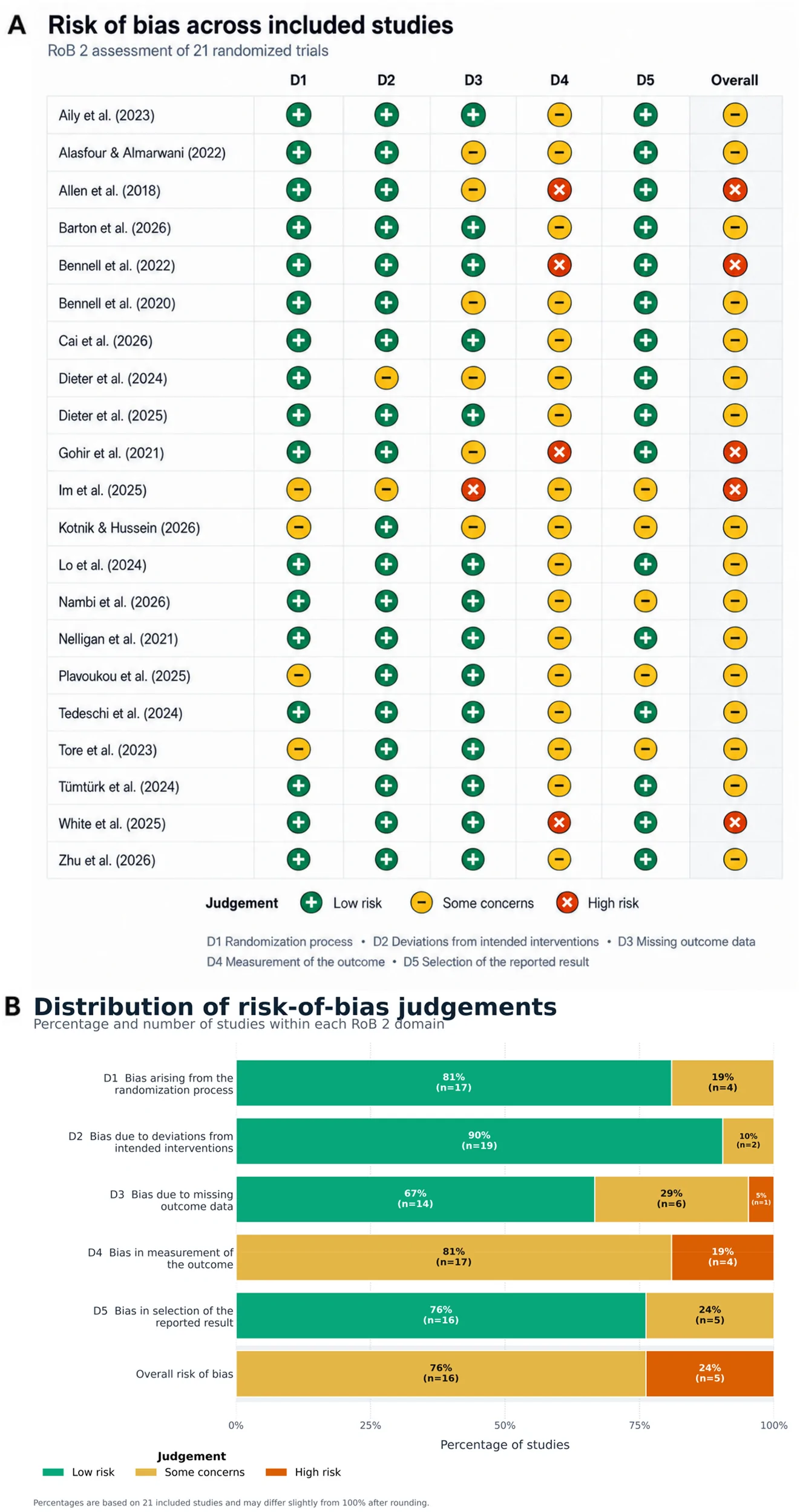
**(A)** Study-level risk-of-bias judgements for the 21 included randomised controlled trials. **(B)** Distribution of risk-of-bias judgements across the five RoB 2 domains and overall. D1, bias arising from the randomisation process; D2, bias due to deviations from intended interventions; D3, bias due to missing outcome data; D4, bias in measurement of the outcome; D5, bias in selection of the reported result.

### 3.4 WOMAC pain

#### 3.4.1 Network structure

The WOMAC pain network included 8 RCTs, 943 participants, and 17 randomised arms, covering all seven intervention nodes and forming eight direct comparisons; Allen 2018 was a three-arm trial. The nodes and direct comparisons formed a single connected network, allowing the simultaneous integration of direct and indirect evidence. The C2–D2 comparison was informed by three trials and therefore had relatively more replicated direct evidence, whereas most other comparisons were supported by a single trial. The D3 node was informed by only one trial involving 21 participants and was therefore sparsely represented in the network. In the network plot, node size was proportional to the sample size assigned to each node, and line thickness reflected the number of trials providing direct evidence for each comparison. The WOMAC pain network is shown in Figure 3A.

**Figure 3:**
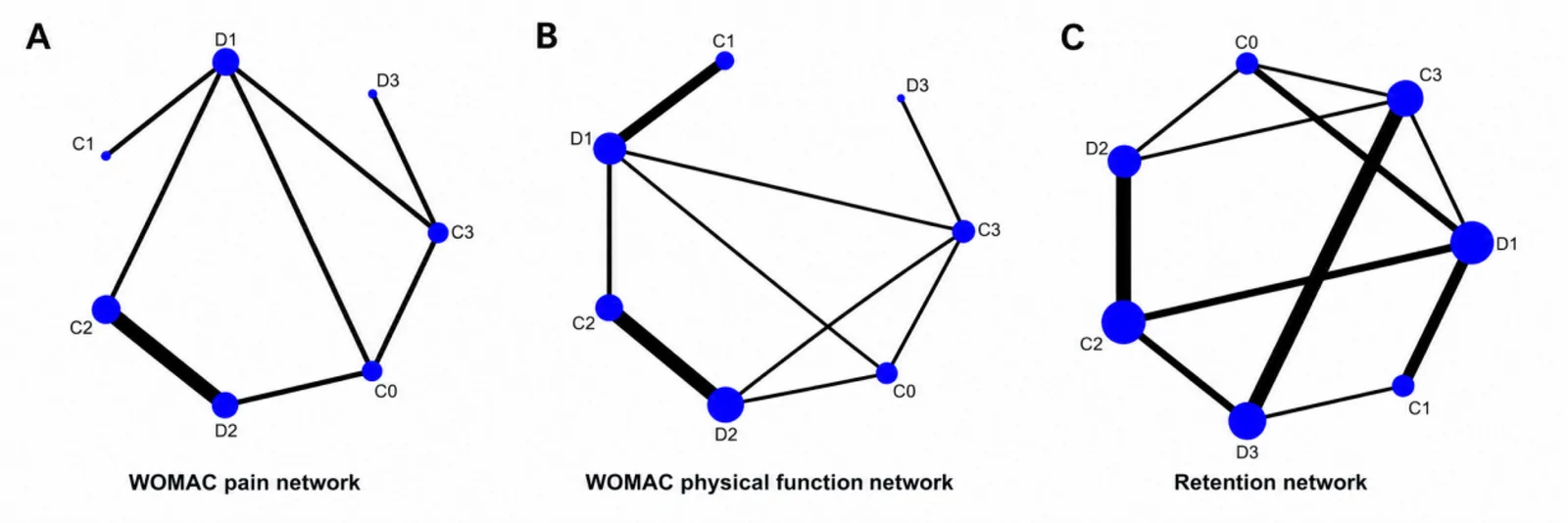
Network plots for the three outcomes: **(A)** WOMAC pain; **(B)** WOMAC physical function; and **(C)** retention at the end of the intervention.

#### 3.4.2 Treatment ranking

In exploratory ranking analyses for WOMAC pain, D3 had the highest SUCRA value (88.7%) and a mean rank of 1.7, followed by D2 (SUCRA 74.5%; mean rank 2.5) and D1 (SUCRA 66.4%; mean rank 3.0). C0 had the lowest SUCRA value (10.2%) and a mean rank of 6.4. Because D3 was supported by only one trial involving 21 participants and its effect estimate was imprecise, this ranking was considered highly uncertain and was not used to support clinical prioritisation. The cumulative rank-probability curves in Figure 4A are presented to show ranking uncertainty rather than to identify a preferred treatment. The complete exploratory ranking metrics for WOMAC pain are presented in Table 2.

**Table 2:** Exploratory treatment ranking metrics for WOMAC pain.

Treatment	SUCRA	PrBest	Mean rank
C0	10.2	0.0	6.4
C1	36.4	1.6	4.8
C2	38.9	1.1	4.7
C3	34.9	0.1	4.9
D1	66.4	5.1	3.0
D2	74.5	18.8	2.5
D3	88.7	73.3	1.7

SUCRA and PrBest are expressed as percentages. PrBest, probability of being ranked first; SUCRA, surface under the cumulative ranking curve. Rankings are exploratory, do not incorporate certainty of evidence, and should be interpreted together with the full cumulative rank-probability distributions and network sparsity.

**Figure 4:**
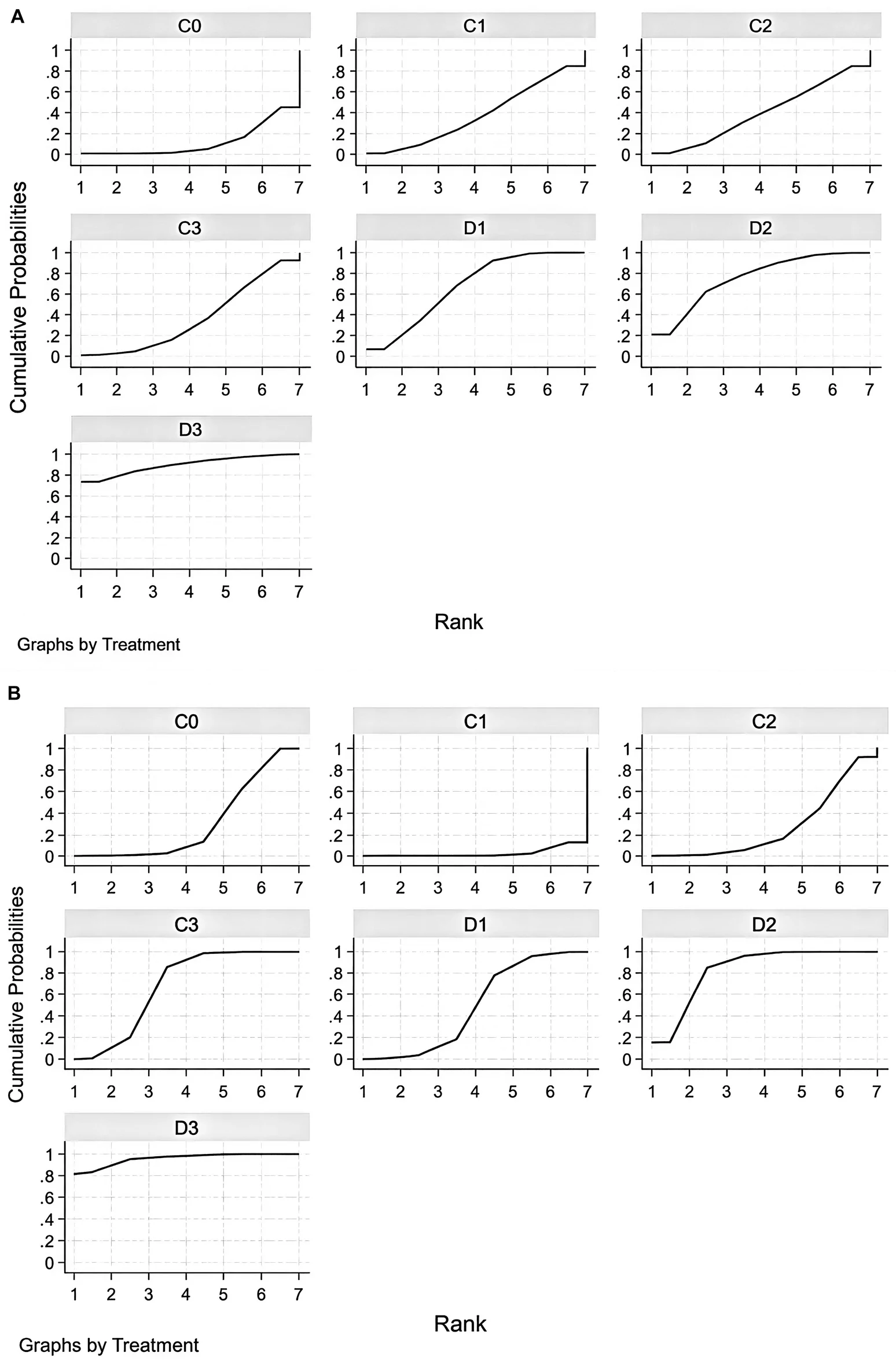
**(A)** Treatment ranking for WOMAC pain. **(B)** Treatment ranking for WOMAC physical function.

#### 3.4.3 Relative effects from the network meta-analysis

Across the three mechanism-matched comparisons, all point estimates favoured the digitally supported nodes, but each comparison used a different comparator and should be interpreted separately. D1 vs. C1 was g = 0.15 (95% CI −0.12 to 0.42), D2 vs. C2 was g = 0.21 (95% CI −0.06 to 0.47), and D3 vs. C3 was g = 0.50 (95% CI −0.12 to 1.12). All three confidence intervals included the null value, and the D3 estimate was particularly imprecise. These standardised effects are not interpreted against a WOMAC-specific minimal clinically important difference.

With C0 as the reference, the network estimates for D1 and D2 were g = 0.30 (95% CI 0.03 to 0.58) and g = 0.38 (95% CI 0.03 to 0.73), respectively, and neither confidence interval included zero. D3 had a larger point estimate relative to C0, but the confidence interval was wide and included the null value (g = 0.65, 95% CI −0.03 to 1.33), reflecting the limitation of evidence from a single small trial. Overall, point estimates for most digitally supported nodes favoured pain improvement, although the mechanism-matched comparisons remained subject to varying degrees of uncertainty. All 21 pairwise comparisons between nodes are presented in the league table and Supplementary Materials. The complete WOMAC pain league table is provided in Supplementary Table S3, and the three mechanism-matched comparisons are shown in Figure 5A.

**Figure 5:**
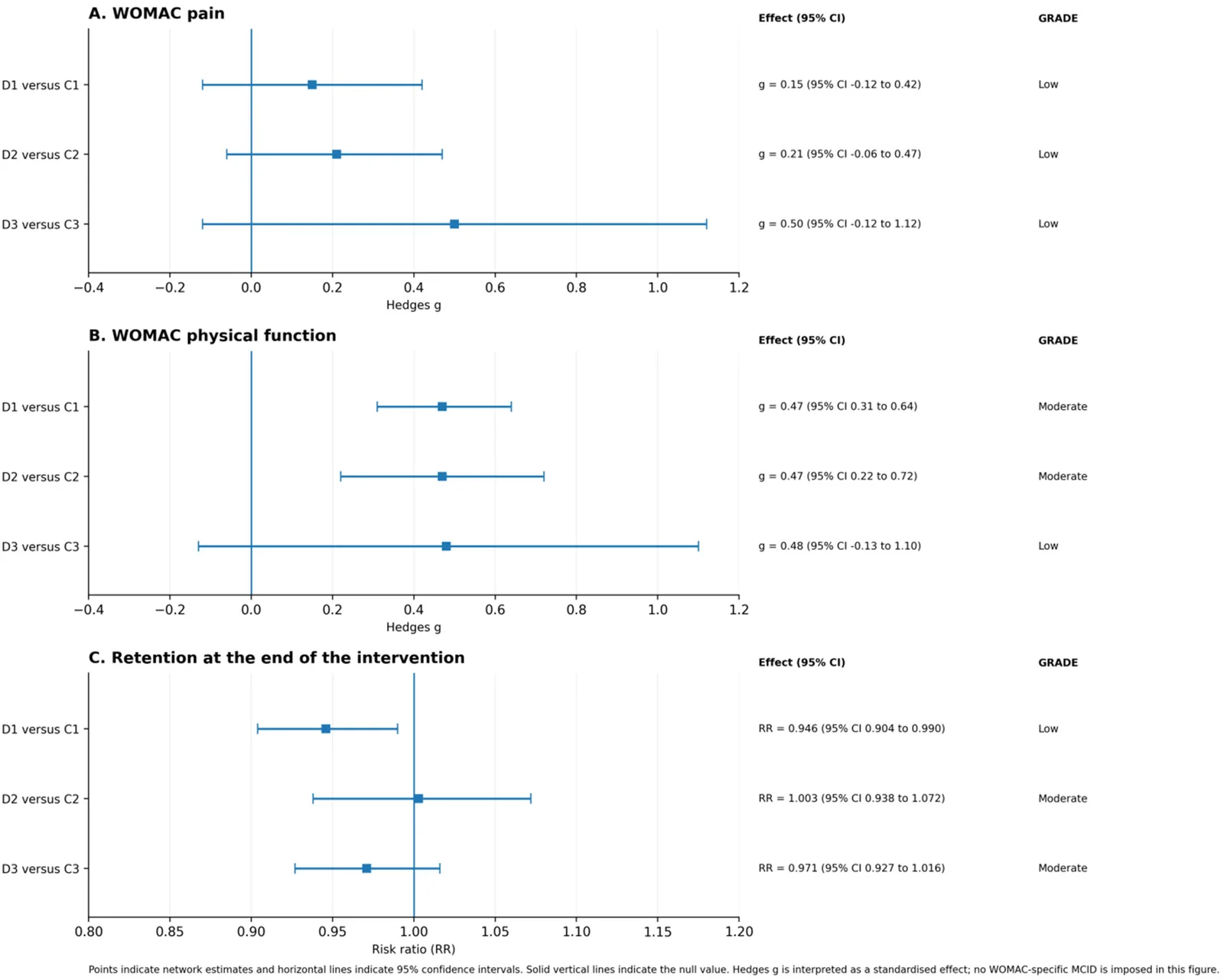
Key mechanism-matched network estimates for **(A)** WOMAC pain, **(B)** WOMAC physical function, and **(C)** retention. Hedges g is presented as a standardised effect; no WOMAC-specific minimal clinically important difference is imposed in this figure.

### 3.5 WOMAC physical function

#### 3.5.1 Network structure

The WOMAC physical function network included 12 RCTs, 1,454 participants, and 25 randomised arms, covering all seven intervention nodes and forming nine direct comparisons; Allen 2018 was a three-arm trial. Compared with the WOMAC pain network, this network additionally included a direct comparison between C3 and D2 and formed a single connected component. D1 had the largest sample size, comprising 449 participants. D3 remained a sparse node, supported by only one trial involving 21 participants. The D2–C2 comparison was informed by four trials, and the D1–C1 comparison by three trials, representing the comparisons with relatively more replicated direct evidence within the network. The WOMAC physical function network is shown in Figure 3B.

#### 3.5.2 Treatment ranking

In exploratory ranking analyses for WOMAC physical function, D3 had the highest SUCRA value (95.6%) and a mean rank of 1.3, followed by D2 (SUCRA 82.4%; mean rank 2.1) and C3 (SUCRA 67.1%; mean rank 3.0). D1 had a SUCRA value of 49.0% and a mean rank of 4.1, whereas C1 had the lowest SUCRA value (2.0%) and a mean rank of 6.9. Because D3 was represented by only one 21-participant intervention arm and the D3 vs. C3 confidence interval was wide, its apparent ranking advantage was considered highly uncertain. Figure 4B is therefore interpreted as a distribution of ranking probabilities rather than evidence of treatment superiority. The complete exploratory ranking metrics for WOMAC physical function are presented in Table 3.

**Table 3:** Exploratory treatment ranking metrics for WOMAC physical function.

Treatment	SUCRA	PrBest	Mean rank
C0	28.3	0.0	5.3
C1	2.0	0.0	6.9
C2	25.5	0.0	5.5
C3	67.1	0.8	3.0
D1	49.0	0.3	4.1
D2	82.4	14.9	2.1
D3	95.6	84.0	1.3

SUCRA and PrBest are expressed as percentages. PrBest, probability of being ranked first; SUCRA, surface under the cumulative ranking curve. Rankings are exploratory, do not incorporate certainty of evidence, and should be interpreted together with the full cumulative rank-probability distributions and network sparsity.

#### 3.5.3 Relative effects from the network meta-analysis

For WOMAC physical function, the comparison-specific network estimates were D1 vs. C1: g = 0.47 (95% CI 0.31 to 0.64), D2 vs. C2: g = 0.47 (95% CI 0.22 to 0.72), and D3 vs. C3: g = 0.48 (95% CI −0.13 to 1.10). The confidence intervals for D1 vs. C1 and D2 vs. C2 excluded the null, whereas the D3 vs. C3 interval was wide and included the null. Because these comparisons used different comparator nodes and represented different multicomponent causal contrasts, similarity in their point estimates should not be used to infer equivalence between digital strategies or the independent contribution of asynchronous or synchronous professional support.

With C0 as the reference, the network estimates were D1 vs. C0: g = 0.16 (95% CI −0.10 to 0.43), D2 vs. C0: g = 0.43 (95% CI 0.14 to 0.73), D3 vs. C0: g = 0.77 (95% CI 0.10 to 1.43), and C3 vs. C0: g = 0.29 (95% CI 0.03 to 0.54). These estimates describe standardised relative effects within the network and should not be interpreted as WOMAC-point clinical thresholds. All 21 relative effects between nodes are presented in the league table and supplementary materials. The complete WOMAC physical function league table is provided in Supplementary Table S4, and the three mechanism-matched comparisons are shown in Figure 5B.

### 3.6 Retention

#### 3.6.1 Network structure

The retention network included 20 RCTs, 2,189 participants, and 41 randomised arms, covering all seven intervention nodes and forming 11 direct comparisons; Allen 2018 was a three-arm trial. The nodes and direct comparisons formed a single connected network, allowing the simultaneous integration of direct and indirect evidence. Compared with the two primary-outcome networks, the retention network included a larger number of studies and comparisons, although the amount of evidence remained unevenly distributed across nodes. In the network plot, node size reflected the number of randomised participants assigned to each node, and line thickness indicated the number of trials providing direct evidence for each comparison. The retention network is shown in Figure 3C.

#### 3.6.2 Relative effects from the network meta-analysis

The retention estimates for all three mechanism-matched comparisons were close to RR = 1. Retention was slightly lower with self-directed digital exercise than with minimal-intensity digital education (D1 vs. C1: RR = 0.946, 95% CI 0.904 to 0.990). The difference between digitally delivered exercise with asynchronous professional support and non-digital self-directed exercise was small (D2 vs. C2: RR = 1.003, 95% CI 0.938 to 1.072). The estimate for synchronous or hybrid professional support vs. face-to-face professionally supervised exercise was also close to no difference (D3 vs. C3: RR = 0.971, 95% CI 0.927 to 1.016). Retention here represents completion of the scheduled assessment and should not be interpreted as exercise adherence, sustained engagement, or acceptability.

The estimates for all three retention comparisons were close to RR = 1. The prespecified 0.83 to 1.20 values are reported only as contextual boundaries and not as validated thresholds for adherence or acceptability. Local direct–indirect inconsistency was identified for D1–C1, and this comparison should therefore be interpreted cautiously. The complete retention league table is provided in Supplementary Table S5, and the three mechanism-matched comparisons are shown in Figure 5C.

### 3.7 Network heterogeneity and inconsistency

In the WOMAC physical function network, the consistency model indicated that the common between-study heterogeneity variance was close to zero (*τ*^2^ ≈ 0.000). The global joint test for inconsistency did not indicate a clear conflict between direct and indirect evidence (*χ*^2^ = 0.21, df = 2, *P* = 0.901). Seven comparisons were assessed using node-splitting analyses, with local *P* values ranging from 0.675 to 0.997. No clear direct–indirect disagreement was detected for C0–C3, C0–D1, C0–D2, C1–D1, C2–D1, C2–D2, or C3–D1. However, the indirect estimate for C1–D1 had a large standard error, suggesting that some indirect evidence pathways were sparse. Accordingly, these findings should not be interpreted as demonstrating complete consistency across the network.

In the WOMAC pain network, the common between-study heterogeneity variance was also close to zero (*τ*^2^ ≈ 0.000). The global joint test for inconsistency did not yield a reportable statistic; therefore, local inconsistency was assessed primarily using node-splitting analyses. Eight comparisons were evaluated, with local *P* values ranging from 0.679 to 0.994, but the absence of a small *P* value was not informative for the sparsest pathways. In particular, the indirect estimates for C1–D1 and C3–D3 had standard errors greater than 63 and were essentially uninformative, demonstrating that mathematical connectivity did not provide clinically useful indirect evidence for these comparisons. Direct, indirect, and network estimates are reported side by side in Supplementary Tables S6 and S13.

In the retention network, the consistency model estimated a small common heterogeneity variance (*τ*^2^ ≈ 0.00022). The global inconsistency test did not indicate a clear overall conflict (*χ*^2^ = 7.61, df = 5, *P* = 0.179), but node-splitting identified local disagreement for C1–D1 and C1–D3 (both *P* = 0.014). Direct C1–D1 evidence came from Bennell et al. (64), Nelligan et al. (71), and Zhu et al. (72), whereas the direct C1–D3 comparison came from White et al. (41). Because the retention network contained multiple overlapping indirect paths, the disagreement could not be attributed uniquely to a single loop or trial. Network estimates involving C1 should therefore be interpreted cautiously. Direct, indirect, and network estimates for the affected comparisons are reported side by side in Supplementary Tables S6 and S13.

### 3.8 Sensitivity and influence analyses

In leave-one-out analyses of WOMAC pain, estimates for D2 vs. C2 ranged from g = 0.16 to 0.36 and all 95% confidence intervals included zero. The point estimate increased after exclusion of Kotnik et al. (38) and decreased after exclusion of Tümtürk et al. (37); exclusion of Lo et al. (36) produced only minor changes. D1 vs. C1 could not be estimated after removal of Bennell et al. (64), and D3 vs. C3 could not be estimated after exclusion of Plavoukou et al. (40), demonstrating dependence on critical direct evidence. When the five trials at overall high risk of bias were excluded, the WOMAC pain network fragmented into disconnected components; D1 vs. C1 was no longer estimable as a network comparison. In the remaining C2–D1–D2 component, D2 vs. C2 was g = 0.17 (95% CI −0.13 to 0.46), while D3 vs. C3 remained the single direct-trial estimate of g = 0.50 (95% CI −0.12 to 1.12). Thus, the high-risk-of-bias sensitivity analysis reduced rather than strengthened the evidential connectivity of the pain network.

The leave-one-out WOMAC physical-function estimates were relatively stable: D1 vs. C1 ranged from g = 0.46 to 0.49 and D2 vs. C2 from g = 0.38 to 0.67. The D2-vs.-C2 estimate increased after exclusion of Kotnik et al. (38) and decreased after exclusion of Alasfour and Almarwani (61). D3 vs. C3 could not be estimated after exclusion of Plavoukou et al. (40). In the *post hoc* analysis excluding the five trials at overall high risk of bias, the remaining functional network retained the key mechanism-matched comparisons (although C0 was no longer represented): D1 vs. C1 was g = 0.49 (95% CI 0.28 to 0.69), D2 vs. C2 was g = 0.44 (95% CI 0.17 to 0.72), and D3 vs. C3 was g = 0.48 (95% CI −0.13 to 1.10). These results did not remove the uncertainty surrounding D3 and should not be interpreted as a low-risk-only analysis because all remaining trials still had at least some concerns.

The leave-one-out findings for retention similarly showed no clear reversal in the direction of effect. D1 vs. C1 estimates ranged from RR = 0.93 to 0.96; after exclusion of White et al. (41) or Zhu et al. (72), the 95% confidence intervals included 1. D2 vs. C2 ranged from RR = 0.97 to 1.02, and D3 vs. C3 from RR = 0.96 to 0.99, with all confidence intervals including 1. Bennell et al. (64), Kotnik et al. (38), Tedeschi et al. (43), and White et al. (41) had relatively large numerical influence on selected comparisons but did not justify automatic exclusion. In the *post hoc* alternative-node analysis, plausible reclassification of Kotnik 2026 and, for retention, the Nambi 2026 control did not materially change D1 vs. C1 but altered selected D2- and D3-related estimates, particularly for the WOMAC outcomes. This finding indicates that estimates involving D3 are sensitive to plausible node definitions and reinforces the need for cautious interpretation. All WOMAC inputs in the frozen primary datasets were change scores, so a separate change-vs.-endpoint sensitivity analysis was not applicable. Full alternative-node and high-risk-of-bias sensitivity estimates are provided in Supplementary Table S12.

### 3.9 Small-study effects and publication bias

The WOMAC pain network included only eight RCTs, which was insufficient to support a reliable regression-based assessment of small-study effects. Therefore, a *post hoc* comparison-adjusted funnel plot is presented in the supplementary materials solely as an exploratory visualisation, without formal statistical inference regarding its symmetry.

The WOMAC physical function network included 12 RCTs. Study estimates in the funnel plot were generally distributed around the zero line, and the exploratory regression did not indicate a clear signal of small-study effects (t = 1.299, df = 10, *P* = 0.223). The retention network included 20 RCTs, and the study estimates similarly showed no clear systematic asymmetry. The regression result was t = −0.161, df = 18, and *P* = 0.874. These findings indicate only that no clear small-study effects were detected given the current number of studies and network structures; they do not exclude the possibility of publication bias. Trial registration completeness, selective outcome reporting, and potentially unpublished studies should also be considered. The comparison-adjusted funnel plots for WOMAC pain, WOMAC physical function, and retention are shown in Supplementary Figure S4A–C. The WOMAC pain plot is presented for exploratory visualisation only.

### 3.10 GRADE certainty of evidence

The WOMAC pain, physical-function, and retention networks comprised 8 RCTs (*N* = 943), 12 RCTs (*N* = 1,454), and 20 RCTs (*N* = 2,189), respectively; these are network-level totals and are not repeated as if they directly informed every specific comparison. Table 4 instead reports the number of trials and participants providing direct evidence for each key mechanism-matched comparison and characterises the availability and precision of indirect evidence. Across all prespecified comparisons, GRADE certainty ranged from moderate to very low; among the key comparisons in Table 4, certainty ranged from moderate to low. For WOMAC pain, certainty was low for D1 vs. C1, D2 vs. C2, and D3 vs. C3 because of study limitations and imprecision; the point estimates favoured the digitally supported nodes, but all confidence intervals included the null. Extremely imprecise indirect evidence for D1 vs. C1 and D3 vs. C3 did not increase confidence in those network estimates.

**Table 4:** GRADE certainty of evidence for key mechanism-matched network comparisons, with direct and indirect evidence structure shown explicitly.

Outcome	Comparison	Direct/indirect evidence	Risk of bias	Inconsistency	Indirectness	Imprecision	Publication bias	Certainty of evidence
WOMAC pain	D1 versus C1 g = 0.15 (95% CI −0.12 to 0.42)	Direct: 1 RCT; 212 outcome participants. Indirect: present but essentially uninformative (SE ≈ 63.27).	Serious	Not serious	Not serious	Serious^a^	Not formally assessed (network k < 10)	Low ⊕⊕○○
WOMAC pain	D2 versus C2 g = 0.21 (95% CI −0.06 to 0.47)	Direct: 3 RCTs; 204 outcome participants. Indirect: available (SE ≈ 0.44).	Serious	Not serious	Not serious	Serious^a^	Not formally assessed (network k < 10)	Low ⊕⊕○○
WOMAC pain	D3 versus C3 g = 0.50 (95% CI −0.12 to 1.12)	Direct: 1 RCT; 42 outcome participants. Indirect: present but essentially uninformative (SE ≈ 63.23).	Serious	Not serious	Not serious	Serious^a^	Not formally assessed (network k < 10)	Low ⊕⊕○○
WOMAC physical function	D1 versus C1 g = 0.47 (95% CI 0.31 to 0.64)	Direct: 3 RCTs; 588 outcome participants. Indirect: present but essentially uninformative (SE ≈ 36.50).	Serious	Not serious	Not serious	Not serious	No clear signal in exploratory diagnostics	Moderate ⊕⊕⊕○
WOMAC physical function	D2 versus C2 g = 0.47 (95% CI 0.22 to 0.72)	Direct: 4 RCTs; 239 outcome participants. Indirect: available (SE ≈ 0.41).	Serious	Not serious	Not serious	Not serious	No clear signal in exploratory diagnostics	Moderate ⊕⊕⊕○
WOMAC physical function	D3 versus C3 g = 0.48 (95% CI −0.13 to 1.10)	Direct: 1 RCT; 42 outcome participants. No independent informative indirect estimate because D3 was a leaf node in this outcome network.	Serious	Not serious	Not serious	Serious^a^	No clear signal in exploratory diagnostics	Low ⊕⊕○○
Retention at the end of the intervention	D1 versus C1 RR = 0.946 (95% CI 0.904 to 0.990)	Direct: 3 RCTs; 596 participants. Indirect: available; local inconsistency *P* = 0.014.	Serious	Serious^b^	Not serious	Not serious	No clear signal in exploratory diagnostics	Low ⊕⊕○○
Retention at the end of the intervention	D2 versus C2 RR = 1.003 (95% CI 0.938 to 1.072)	Direct: 4 RCTs; 277 participants. Indirect: available; no clear local disagreement.	Serious	Not serious	Not serious	Not serious	No clear signal in exploratory diagnostics	Moderate ⊕⊕⊕○
Retention at the end of the intervention	D3 versus C3 RR = 0.971 (95% CI 0.927 to 1.016)	Direct: 4 RCTs; 176 participants. Indirect: available; node-splitting *P* = 0.074.	Serious	Not serious	Not serious	Not serious	No clear signal in exploratory diagnostics	Moderate ⊕⊕⊕○

Network-level totals are reported in the Results but are not used as the per-comparison evidence count. “Direct” denotes trials containing the comparison itself. “Indirect” describes evidence from the remainder of the network and its precision based on node-splitting. Certainty ratings were derived for the network estimate using study limitations, inconsistency, indirectness/transitivity, imprecision, and publication-bias considerations.

a Downgraded one level for imprecision because the 95% CI included the null and/or encompassed materially different plausible effects.

b Downgraded one level for local direct–indirect inconsistency (node-splitting *P* = 0.014).

For WOMAC physical function, the network estimates were g = 0.47 (95% CI 0.31 to 0.64) for D1 vs. C1, g = 0.47 (95% CI 0.22 to 0.72) for D2 vs. C2, and g = 0.48 (95% CI −0.13 to 1.10) for D3 vs. C3. The confidence intervals for D1 vs. C1 and D2 vs. C2 excluded the null and were comparatively precise, supporting moderate-certainty evidence after downgrading for study limitations. This should not be interpreted as proof that the effects exceeded a validated WOMAC-specific clinical-importance threshold. D3 vs. C3 was supported directly by one small trial, had no independent informative indirect evidence, and remained low certainty because of imprecision and study limitations.

For retention, the certainty of evidence for D1 vs. C1 was low because study limitations were compounded by local direct–indirect inconsistency. Certainty was moderate for D2 vs. C2 and D3 vs. C3. These ratings apply to the network estimates of trial completion and should not be interpreted as evidence about exercise adherence or acceptability. Detailed domain judgements for all prespecified comparisons are provided in Supplementary Table S8.

## 4 Discussion

### 4.1 Principal findings

This study included 21 randomised controlled trials and grouped interventions into seven prespecified delivery/support nodes. The most clearly estimated functional findings were comparison-specific: D1 vs. C1 and D2 vs. C2 each had confidence intervals that excluded the null, with moderate-certainty evidence, whereas D3 vs. C3 remained imprecise and was supported directly by a single small trial. Because D1 vs. C1, D2 vs. C2, and D3 vs. C3 represent different multicomponent causal contrasts, their effect magnitudes should not be compared as if they quantified increasing doses of the same intervention. In particular, the results do not isolate the effect of digital delivery itself or the independent contribution of professional support.

All three mechanism-matched WOMAC pain point estimates favoured the digitally supported nodes, but all confidence intervals included the null and certainty was low. The pain network was mathematically connected but clinically sparse, and some indirect paths were essentially uninformative. Retention estimates were generally close to RR = 1 and describe trial assessment completion rather than sustained exercise participation. Local inconsistency for D1 vs. C1 further limits interpretation of that retention comparison. Overall, the data provide the strongest comparison-specific signal for patient-reported physical function, while the incremental effects on pain, the independent value of professional support, and differences in trial completion remain uncertain.

Importantly, D1, D2, and D3 were compared with digital education, non-digital self-directed exercise, and face-to-face professionally supervised exercise, respectively. The three effect estimates were therefore not obtained against a common comparator. Consequently, similar or progressively larger effect estimates should not be interpreted as evidence that the different digital-support approaches are equally effective, nor do they establish a linear relationship whereby greater digital-support intensity produces greater treatment effects. D3 ranked highest for both WOMAC outcomes; however, the evidence supporting this node was sparse, and the ranking did not account for risk of bias, imprecision, or GRADE certainty. D3 should therefore not be regarded as the preferred intervention solely on the basis of its ranking.

### 4.2 Interpretation and comparison with previous evidence

Digital platforms can provide repeatedly accessible exercise demonstrations, training plans, reminders, self-monitoring, progress tracking, and remote communication (39, 42, 44–47). Asynchronous professional support can also provide non-real-time advice or prescription adjustment (37, 48–50). These features are plausible mechanisms through which multicomponent digital programmes might influence exercise implementation; however, they were not isolated or experimentally compared within this network meta-analysis. Because the nodes differed simultaneously in exercise content, digital delivery, automated feedback, behavioural support, professional contact, and comparator intensity, these mechanisms should be regarded as explanatory hypotheses rather than causal pathways demonstrated by the network.

The D1-vs.-C1 and D2-vs.-C2 estimates should be interpreted independently. D1 vs. C1 compares a structured self-directed digital exercise package with digital education, whereas D2 vs. C2 compares a digitally delivered exercise package containing asynchronous professional support with non-digital self-directed exercise. The similarity of the two point estimates does not quantify the contribution of asynchronous professional support and does not demonstrate equivalence between D1 and D2. Likewise, D3 vs. C3 addresses a different contrast—synchronous or hybrid digital support vs. face-to-face professional supervision—and was directly supported by only one small WOMAC trial. The *post hoc* alternative-node sensitivity analysis further showed that D3 estimates were sensitive to plausible classification choices.

The effects on pain were more uncertain than those on physical function. Improvements in function may not necessarily translate into parallel reductions in pain because pain is influenced not only by muscle strength and physical capacity but also by natural symptom fluctuations, treatment expectations, psychological status, previous pain experiences, medication use, and other co-interventions (52–54). The pain network included only eight RCTs, most direct comparisons were supported by a single study, and some indirect evidence pathways were extremely sparse. These limitations further reduced the precision of the effect estimates and the ability of inconsistency tests to detect disagreement. The present findings should therefore not be described as showing that digital support is “ineffective for pain”; rather, the available evidence remains insufficient to determine its incremental effect on pain relative to mechanism-matched comparators.

Retention estimates were generally close to no difference, suggesting that the form of digital technology or the degree of professional support may not be the sole determinant of study completion. Completion of the end-of-intervention assessment may also be influenced by study burden, changes in symptoms, digital literacy, intervention preferences, and relationships with healthcare professionals. Retention must be distinguished from exercise adherence. Retention indicates only whether participants remained in the study until the prespecified assessment and does not reflect the number of exercise sessions completed, the dose achieved, or the extent to which the prescribed programme was followed. Accordingly, the present study cannot determine from retention data which delivery approach is more conducive to sustained exercise participation.

Previous systematic reviews have generally combined interventions delivered through mobile applications, websites, text messaging, videos, sensors, and remote professional guidance into broad categories of digital health or telerehabilitation and have primarily examined whether digital interventions are effective compared with usual care or conventional rehabilitation (55–57). Some reviews have suggested that digital interventions improve pain and function, whereas others have not identified a clear advantage for physical function. These differences may reflect variation in the included populations, outcome measures, comparator intensity, and the pooling of distinct support mechanisms (58–60). The present study further classified interventions according to exercise delivery, feedback, and professional involvement and prioritised mechanism-matched comparisons. This shifted the research question from whether digital interventions are effective to whether different digital-support mechanisms provide incremental value beyond comparable underlying interventions. The findings suggest that functional benefits may exist, but there is currently insufficient evidence to conclude that greater synchronous professional involvement necessarily produces larger effects.

### 4.3 Clinical and research implications

The present network meta-analysis does not identify patient subgroups who should preferentially receive self-directed, asynchronously supported, or synchronously supported digital care. The results provide comparison-specific evidence about particular multicomponent intervention packages, not validated patient-level treatment-selection rules. Factors such as digital literacy, access to devices and connectivity, self-management capacity, availability of professional resources, and patient preference remain clinically relevant when choosing a delivery model, but they were not tested as effect modifiers in this study. Statements that particular patients are better suited to D1 or D2 should therefore be treated as hypotheses for future stratified trials rather than recommendations supported by the present network.

The available evidence does not support routinely prioritising synchronous or hybrid digital support over face-to-face supervised exercise, nor does it support the wholesale replacement of conventional rehabilitation with digital interventions. The functional effect and high ranking of D3 were derived primarily from a single small trial, and its clinical superiority has not been reliably established. For pain outcomes, digital exercise may be more appropriately implemented as one component of comprehensive management, alongside patient education, symptom monitoring, weight management, and pharmacological treatment when indicated, rather than being regarded as a stand-alone strategy with a confirmed analgesic effect. Retention should likewise not be used as the sole basis for selecting a digital-support approach because it does not reflect actual exercise performance.

Future research should prioritise adequately powered head-to-head trials comparing synchronous or hybrid digital support with face-to-face professionally supervised exercise and should increase the number of direct comparisons among D1, D2, and D3 to reduce the dependence of key conclusions on indirect evidence and individual studies. Trials should clearly specify the digital-support mechanism, mode of professional contact, frequency of feedback, and co-interventions at the registration or protocol stage. They should also standardise the scoring direction, assessment time points, and effect-reporting formats of WOMAC subscales. In addition to outcomes measured at the end of the intervention, future studies should report long-term pain and functional outcomes, actual exercise completion, platform use, reasons for withdrawal, adverse events, professional input, and cost-effectiveness to determine whether short-term functional benefits can be sustained and translated into implementable models of clinical service delivery.

### 4.4 Strengths and limitations

A design feature of this study was the use of a mechanism-based node framework rather than classification solely by technology brand or device type. This improved transparency about exercise delivery, feedback, and professional contact, but it does not establish clinical homogeneity within nodes or guarantee transitivity across comparisons. The framework therefore represents both an attempt to improve interpretability and an important source of modelling uncertainty. Other strengths include separate networks for WOMAC pain, WOMAC physical function, and retention; explicit handling of shared comparators in multi-arm trials; and reporting of network effects, inconsistency diagnostics, influence analyses, ranking uncertainty, and GRADE certainty.

This study has several important limitations. First, the WOMAC pain and physical-function networks included only 8 and 12 RCTs, respectively, and most direct comparisons were supported by few trials; D3 was represented by only one 21-participant intervention arm in both WOMAC networks. Second, no included trial was at overall low risk of bias and five were at high risk; excluding high-risk trials fragmented the pain network. Third, node membership necessarily collapsed heterogeneous multicomponent interventions. The analysis cannot separate the effects of digital technology from differences in exercise prescription, dose/progression, behavioural support, therapist contact, education, or other co-interventions. The alternative-node sensitivity analysis demonstrated that estimates involving D3 could change materially under plausible classification choices. Fourth, potential effect modifiers were incompletely and unevenly reported, so transitivity could be assessed only descriptively and remained uncertain. Fifth, participants could not generally be blinded and WOMAC outcomes were patient reported, creating susceptibility to expectations and measurement bias.

In addition, the retention network showed local direct–indirect inconsistency involving C1, while some indirect pain estimates had standard errors greater than 63 and were essentially uninformative. Leave-one-out analyses identify dependence on individual trials but cannot resolve all structural assumptions of the network. Although high-risk-of-bias and alternative-node sensitivity analyses were conducted, *post hoc* duration- or comparator-intensity cut-points were not imposed because such choices were not prespecified and would further fragment the sparse WOMAC networks. The search was restricted to seven electronic databases plus manual reference-list screening and did not include a systematic search of clinical trial registries or dedicated grey-literature databases; unpublished or difficult-to-locate studies may therefore have been missed. Comparison-adjusted funnel plots cannot exclude publication bias. Finally, retention represents completion of a scheduled study assessment and does not quantify exercise dose, adherence, engagement, or acceptability, and the analyses primarily reflect end-of-intervention or nearest post-intervention effects rather than long-term outcomes.

## 5 Conclusion

This systematic review and network meta-analysis compared seven prespecified multicomponent delivery/support strategies for knee osteoarthritis. For patient-reported physical function, the comparison-specific estimates for D1 vs. C1 and D2 vs. C2 had confidence intervals that excluded the null and were rated moderate certainty; however, these are different causal contrasts and do not establish superiority of digital delivery itself or the independent value of asynchronous professional support. D3 vs. C3 remained uncertain because it was directly supported by one small trial and was sensitive to plausible node reclassification. All included trials had at least some risk-of-bias concerns, and node heterogeneity and residual uncertainty in transitivity limit causal interpretation.

For WOMAC pain, all three mechanism-matched confidence intervals included the null and certainty was low; sparse and in some cases essentially uninformative indirect evidence further limits confidence. Retention estimates were generally close to RR = 1 and should be interpreted only as trial-completion outcomes, with local inconsistency affecting D1 vs. C1. These findings do not support a linear relationship between digital-support intensity and treatment effects and should not be used to prioritise synchronous or hybrid professional support solely on the basis of ranking outputs.

Further adequately powered, preregistered head-to-head trials are needed to compare clearly defined delivery/support mechanisms while holding exercise content and co-interventions as constant as possible. Future studies should report standardised WOMAC outcome data, long-term follow-up, actual exercise adherence and dose, intervention components, resource use, and cost-effectiveness, and should prospectively evaluate whether patient characteristics modify the effect of different digital-support strategies.

## Data Availability

The original contributions presented in the study are included in the article/Supplementary Material, further inquiries can be directed to the corresponding author.
