# Supplementary material for: Comparative effectiveness of exercise and self-management interventions delivered through different modes of digital support on pain and patient-reported physical function in people with knee osteoarthritis: a systematic review and network meta-analysis of randomised controlled trials
Source: Front Med (Lausanne). 2026 Sep 30;13:1956102. doi: 10.3389/fmed.2026.1956102 (PMC13661503; doi:10.3389/fmed.2026.1956102)
Supplement: Supplementary file 1 [file Supplementaryfile1.pdf]

# Supplementary Materials

## Comparative Effectiveness of Exercise and Self-Management Interventions Delivered Through Different Modes of Digital Support on Pain and Patient-Reported Physical Function in People With Knee Osteoarthritis

*Supplementary materials for the systematic review and network meta-analysis of randomised controlled trials*

## Contents


| Item | Content |
|---|---|
| Supplementary Methods | Outcome-data harmonisation and effect-size derivation |
| Supplementary Table S1 | Database search strategies, search coverage, and retrieval counts |
| Supplementary Table S2 | Node definitions, operational assignment algorithm, and arm-level mapping |
| Supplementary Table S3 | Complete WOMAC pain league table |
| Supplementary Table S4 | Complete WOMAC physical-function league table |
| Supplementary Table S5 | Complete retention league table |
| Supplementary Table S6 | Global inconsistency and node-splitting results |
| Supplementary Table S7 | Complete leave-one-out estimates and non-estimable comparisons |
| Supplementary Table S8 | Detailed GRADE judgements and evidence profiles |
| Supplementary Table S9 | Arm-level intervention components and node-defining features |
| Supplementary Table S10 | Frozen study-level WOMAC inputs and derived direct Hedges g estimates |
| Supplementary Table S11 | Descriptive transitivity assessment across direct WOMAC comparisons |
| Supplementary Table S12 | Additional structural and risk-of-bias sensitivity analyses |
| Supplementary Table S13 | Direct, indirect, and network evidence for comparisons highlighted by inconsistency diagnostics |
| Supplementary Figures S1A-C | Leave-one-out plots for WOMAC pain |
| Supplementary Figures S2A-C | Leave-one-out plots for WOMAC physical function |
| Supplementary Figures S3A-C | Leave-one-out plots for retention |
| Supplementary Figures S4A-C | Comparison-adjusted funnel plots |

## Supplementary Methods. Outcome-data harmonisation and effect-size derivation

Continuous WOMAC outcomes were extracted using a prespecified hierarchy. Baseline-adjusted between-group estimates were prioritised when they could be represented on the prespecified standardised scale; otherwise change scores were used, with endpoint values retained as a prespecified fallback. In the frozen WOMAC pain and physical-function datasets used for the final network meta-analyses, every arm-level input was represented as a change score; therefore, the primary networks did not mix change-score and endpoint inputs.

For a change score, negative source values denoted improvement. When the standard deviation of change ($SD_{change}$) was not reported and could not be recovered from convertible statistics, it was calculated from the baseline and follow-up standard deviations using $SD_{change} = \sqrt{SD_{pre}^2 + SD_{post}^2 - 2r \cdot SD_{pre} \cdot SD_{post}}$. A study-specific pre-post correlation was used when recoverable; otherwise the prespecified $r = 0.50$ was used. The $r = 0.50$ assumption was confirmed against the archived analysis log for this revision.

For a two-arm comparison, the pooled change-score standard deviation was $S_p = \sqrt{[(n1-1)SD1^2 + (n0-1)SD0^2]/(n1+n0-2)}$. With source change scores coded so that more negative values indicated greater improvement, Cohen d for the first-listed intervention versus comparator was calculated as $d = (Mean_{change,comparator} - Mean_{change,intervention})/S_p$, so that positive values favoured the first-listed node. Hedges $g = J \cdot d$, where $J = 1 - 3/[4(n1+n0-2)-1]$. The approximate sampling variance was $V_g = J^2 \cdot [(n1+n0)/(n1 \cdot n0) + d^2/(2(n1+n0-2))]$. For the three-arm Allen trial, arm-level effects were retained and covariance due to the shared comparator was handled by the network model rather than treating the contrasts as independent.

When a study-reported adjusted between-group estimate was eligible, its reported uncertainty (SE or CI) was first converted to a standard error where required and then standardised by the corresponding within-study dispersion before applying the Hedges small-sample correction. The final frozen WOMAC networks, however, were represented by the arm-level change-score inputs shown in Supplementary Table S10. Accordingly, a change-score-versus-endpoint sensitivity analysis was not applicable.

Hedges g was used because the included trials used different WOMAC versions, response formats, scoring ranges, and within-study dispersions. The conventional $|g|$ approximately 0.20 benchmark was used only as a descriptive effect-size reference and not as a WOMAC-specific minimal clinically important difference or as the sole basis for GRADE imprecision. GRADE imprecision was reassessed independently by two reviewers during revision; the resulting certainty ratings were unchanged.

## Supplementary Table S1. Database search strategies, search coverage, and retrieval counts

*Seven electronic databases were systematically searched from inception to July 2026. Clinical trial registries (e.g., ClinicalTrials.gov) and dedicated grey-literature databases were not systematically searched. Reference lists of included studies and relevant systematic reviews were screened manually. Trial-registration records were consulted only when needed to clarify methods or missing data for otherwise eligible reports; this was not a systematic registry search.*

| Database | Coverage | Records retrieved |
|---|---|---|
| PubMed | Inception-July 2026 | 874 |
| Web of Science Core Collection | Inception-July 2026 | 159 |
| Embase | Inception-July 2026 | 744 |
| Cochrane Library | Inception-July 2026 | 45 |
| Scopus | Inception-July 2026 | 2361 |
| CINAHL | Inception-July 2026 | 288 |
| Wiley Online Library | Inception-July 2026 | 18 |
| Total | | 4489 |

## PubMed

("Osteoarthritis, Knee"[Mesh] OR "knee osteoarthritis"[tiab] OR "knee osteoarthrosis"[tiab] OR "osteoarthritis of the knee"[tiab] OR "osteoarthrosis of the knee"[tiab] OR "degenerative arthritis of the knee"[tiab] OR gonarthrosis[tiab] OR gonarthroses[tiab] OR gonarthritis[tiab] OR "knee OA"[tiab] OR KOA[tiab] OR (knee[tiab] AND (osteoarthrit*[tiab] OR osteoarthros*[tiab] OR "degenerative arthritis"[tiab]))) AND ("Digital Health"[Mesh] OR "Telemedicine"[Mesh] OR "Telerehabilitation"[Mesh] OR "Internet-Based Intervention"[Mesh] OR "Mobile Applications"[Mesh] OR "Remote Consultation"[Mesh] OR "Remote Patient Monitoring"[Mesh] OR "Wearable Electronic Devices"[Mesh] OR "Text Messaging"[Mesh] OR "Smartphone"[Mesh] OR "Cell Phone"[Mesh] OR "Telephone"[Mesh] OR "Videoconferencing"[Mesh] OR "Internet"[Mesh] OR "Virtual Reality"[Mesh] OR "digital health"[tiab] OR "digital intervention"[tiab] OR "digitally supported"[tiab] OR "digitally delivered"[tiab] OR "digital rehabilitation"[tiab] OR ehealth[tiab] OR mhealth[tiab] OR telehealth[tiab] OR telemedicine[tiab] OR telerehabilitation[tiab] OR "tele-rehabilitation"[tiab] OR "remote rehabilitation"[tiab] OR "remote physiotherapy"[tiab] OR "remote exercise"[tiab] OR "internet-based"[tiab] OR "web-based"[tiab] OR "online exercise"[tiab] OR "mobile application"[tiab] OR "mobile app"[tiab] OR "app-based"[tiab] OR smartphone*[tiab] OR "telephone support"[tiab] OR "text messaging"[tiab] OR videoconferenc*[tiab] OR wearable*[tiab] OR sensor*[tiab] OR "automated feedback"[tiab] OR "real-time feedback"[tiab] OR "virtual reality"[tiab]) AND ("Exercise Therapy"[Mesh] OR "Exercise"[Mesh] OR "Physical Therapy Modalities"[Mesh] OR "Rehabilitation"[Mesh] OR "Self-Management"[Mesh] OR exercis*[tiab] OR "physical activity"[tiab] OR physiotherap*[tiab] OR "physical therapy"[tiab] OR rehabilitat*[tiab] OR self-manag*[tiab] OR "home exercise"[tiab] OR "therapeutic exercise"[tiab] OR "exercise training"[tiab] OR strengthening[tiab] OR "resistance training"[tiab] OR walking[tiab] OR yoga[tiab] OR "tai chi"[tiab] OR "patient education"[tiab]) AND (randomized controlled trial[pt] OR controlled clinical trial[pt] OR randomized[tiab] OR randomised[tiab] OR randomly[tiab] OR trial[tiab] OR groups[tiab]) NOT (animals[mh] NOT humans[mh])

## Web of Science Core Collection

TS=((knee* NEAR/3 osteoarthrit*) OR (knee* NEAR/3 osteoarthros*) OR "osteoarthritis of the knee" OR "knee osteoarthritis" OR "knee OA" OR gonarthr*) AND TS=("digital health" OR "digital intervention" OR "digitally supported" OR "digitally delivered" OR "digital rehabilitation" OR ehealth OR mhealth OR telehealth OR telemedicine OR telerehab* OR "tele-rehabilitation" OR (remote NEAR/3 (rehabilitat* OR physiotherap* OR exercis* OR coach* OR monitor*)) OR (internet NEAR/3 (intervention* OR program* OR exercis* OR rehabilitat*)) OR (web NEAR/3 (based OR deliver* OR intervention* OR program* OR exercis*)) OR (online NEAR/3 (intervention* OR program* OR exercis* OR rehabilitat*)) OR (mobile NEAR/3 (app OR application* OR intervention* OR exercis* OR rehabilitat*)) OR smartphone* OR "app-based" OR telephone OR "text messag*" OR videoconferenc* OR wearable* OR sensor* OR acceleromet* OR "automated feedback" OR "real-time feedback" OR "virtual reality" OR exergam*) AND TS=(exercis* OR "exercise therapy" OR "therapeutic exercise" OR "exercise training" OR physiotherap* OR "physical therapy" OR rehabilitat* OR self-manag* OR "home exercise" OR "physical activity" OR strengthening OR "resistance training" OR walking OR yoga OR "tai chi" OR "patient education") AND TS=(random* OR (controlled NEAR/2 trial*) OR (clinical NEAR/2 trial*) OR placebo* OR (control* NEAR/3 group*) OR (trial* NEAR/3 group*))

## Embase

('knee osteoarthritis'/exp OR 'knee osteoarthritis':ti,ab OR 'knee osteoarthrosis':ti,ab OR 'osteoarthritis of the knee':ti,ab OR gonarthrosis:ti,ab OR 'knee oa':ti,ab) AND ('digital health'/exp OR 'telemedicine'/exp OR 'telerehabilitation'/exp OR 'mobile application'/exp OR 'remote patient monitoring'/exp OR 'wearable device'/exp OR 'text messaging'/exp OR 'videoconferencing'/exp OR 'virtual reality'/exp OR 'digital health':ti,ab OR 'digital intervention':ti,ab OR 'digital rehabilitation':ti,ab OR telehealth:ti,ab OR telemedicine:ti,ab OR telerehabilitation:ti,ab OR 'remote rehabilitation':ti,ab OR 'web-based':ti,ab OR 'online exercise':ti,ab OR 'app-based':ti,ab OR smartphone*:ti,ab OR wearable*:ti,ab OR sensor*:ti,ab OR 'automated feedback':ti,ab OR 'real-time feedback':ti,ab OR 'virtual reality':ti,ab) AND ('exercise'/exp OR 'exercise therapy'/exp OR 'physiotherapy'/exp OR 'rehabilitation'/exp OR 'self management'/exp OR exercis*:ti,ab OR physiotherap*:ti,ab OR rehabilitat*:ti,ab OR self-manag*:ti,ab OR 'home exercise':ti,ab OR 'physical activity':ti,ab OR strengthening:ti,ab OR walking:ti,ab OR yoga:ti,ab OR 'tai chi':ti,ab) AND ('randomized controlled trial'/exp OR 'controlled clinical trial'/exp OR random*:ti,ab OR placebo*:ti,ab OR trial:ti,ab OR 'control group':ti,ab) NOT ('animal'/exp NOT 'human'/exp)

## Cochrane Library

([mh "Osteoarthritis, Knee"] OR "knee osteoarthritis":ti,ab,kw OR "knee osteoarthrosis":ti,ab,kw OR "osteoarthritis of the knee":ti,ab,kw OR gonarthrosis:ti,ab,kw OR "knee OA":ti,ab,kw) AND ([mh "Digital Health"] OR [mh Telemedicine] OR [mh Telerehabilitation] OR [mh "Mobile Applications"] OR [mh "Text Messaging"] OR [mh Videoconferencing] OR [mh "Virtual Reality"] OR "digital health":ti,ab,kw OR "digital rehabilitation":ti,ab,kw OR telehealth:ti,ab,kw OR telerehabilitation:ti,ab,kw OR "remote rehabilitation":ti,ab,kw OR "web-based":ti,ab,kw OR "online exercise":ti,ab,kw OR "app-based":ti,ab,kw OR smartphone*:ti,ab,kw OR wearable*:ti,ab,kw OR sensor*:ti,ab,kw OR "automated feedback":ti,ab,kw OR "real-time feedback":ti,ab,kw) AND ([mh Exercise] OR [mh "Exercise Therapy"] OR [mh Rehabilitation] OR [mh "Self-Management"] OR exercis*:ti,ab,kw OR physiotherap*:ti,ab,kw OR rehabilitat*:ti,ab,kw OR self-manag*:ti,ab,kw OR "home exercise":ti,ab,kw OR "physical activity":ti,ab,kw OR strengthening:ti,ab,kw OR walking:ti,ab,kw OR yoga:ti,ab,kw OR "tai chi":ti,ab,kw)

## Scopus

TITLE-ABS-KEY("knee osteoarthritis" OR "knee osteoarthrosis" OR "osteoarthritis of the knee" OR gonarthrosis OR "knee OA" OR KOA OR (knee W/3 osteoarthrit*) OR (knee W/3 osteoarthros*)) AND TITLE-ABS-KEY("digitally supported exercise" OR "digitally delivered exercise" OR "digital exercise" OR "digital rehabilitation" OR "digital physiotherapy" OR "digital self-management" OR "online exercise" OR "online rehabilitation" OR "internet-based exercise" OR "web-based exercise" OR "mobile exercise" OR "app-based exercise" OR "smartphone-based exercise" OR "remote exercise" OR "remote rehabilitation" OR telehealth OR telemedicine OR telerehabilitation OR telephysiotherapy OR "telephone support" OR videoconferenc* OR "text messaging" OR "mobile application" OR smartphone* OR wearable* OR sensor* OR acceleromet* OR "automated feedback" OR "real-time feedback" OR "virtual reality" OR exergam* OR ((exercis* OR physiotherap* OR rehabilitat* OR "physical activity" OR self-manag* OR "home exercise") AND (digital OR online OR internet OR web OR mobile OR smartphone* OR app OR telehealth OR remote OR virtual OR video OR telephone OR wearable* OR sensor*))) AND TITLE-ABS-KEY(random* OR trial OR RCT OR placebo OR "randomized controlled trial" OR "randomised controlled trial" OR "controlled clinical trial" OR "parallel group" OR "control group" OR "wait-list control" OR "cluster randomized" OR "cluster randomised")

## CINAHL

((MH "Osteoarthritis, Knee+") OR TI ("knee osteoarthritis" OR "knee osteoarthrosis" OR "osteoarthritis of the knee" OR gonarthrosis OR "knee OA") OR AB ("knee osteoarthritis" OR "knee osteoarthrosis" OR "osteoarthritis of the knee" OR gonarthrosis OR "knee OA")) AND ((MH "Telehealth+") OR (MH "Telemedicine+") OR (MH "Mobile Applications+") OR (MH "Videoconferencing+") OR (MH "Wearable Devices+") OR TI ("digital health" OR "digital rehabilitation" OR telehealth OR telerehabilitation OR "remote rehabilitation" OR "web-based" OR "online exercise" OR "app-based" OR smartphone* OR wearable* OR sensor* OR "automated feedback" OR "real-time feedback" OR "virtual reality") OR AB ("digital health" OR "digital rehabilitation" OR telehealth OR telerehabilitation OR "remote rehabilitation" OR "web-based" OR "online exercise" OR "app-based" OR smartphone* OR wearable* OR sensor* OR "automated feedback" OR "real-time feedback" OR "virtual reality")) AND ((MH "Exercise+") OR (MH "Exercise Therapy+") OR (MH "Physical Therapy+") OR (MH "Rehabilitation+") OR (MH "Self Care+") OR TI (exercis* OR physiotherap* OR rehabilitat* OR self-manag* OR "home exercise" OR "physical activity" OR strengthening OR walking OR yoga OR "tai chi") OR AB (exercis* OR physiotherap* OR rehabilitat* OR self-manag* OR "home exercise" OR "physical activity" OR strengthening OR walking OR yoga OR "tai chi")) AND ((MH "Randomized Controlled Trials") OR (MH "Random Assignment") OR TI (random* OR trial OR placebo*) OR AB (random* OR trial OR placebo* OR "control group"))

## Wiley Online Library

("knee osteoarthritis" OR "knee osteoarthrosis" OR "osteoarthritis of the knee" OR gonarthrosis OR "knee OA") AND ("digital health" OR "digital rehabilitation" OR telehealth OR telemedicine OR telerehabilitation OR "remote rehabilitation" OR "web-based" OR "online exercise" OR "mobile app" OR "app-based" OR smartphone OR wearable OR sensor OR videoconference OR "text messaging" OR "automated feedback" OR "real-time feedback" OR "virtual reality") AND (exercise OR "exercise therapy" OR physiotherapy OR rehabilitation OR "self-management" OR "home exercise" OR "physical activity" OR strengthening OR walking OR yoga OR "tai chi") AND (random* OR trial OR "controlled trial" OR RCT)

# Supplementary Table S2. Node definitions, operational assignment algorithm, and arm-level mapping

| Node | Operational definition | Network role |
|---|---|---|
| C0 | Usual care, waiting-list control, or no study-prescribed structured exercise | Clinical reference |
| C1 | Minimal-intensity digital education without a structured digital exercise prescription | Digital-attention control |
| C2 | Non-digital self-directed exercise without ongoing professional supervision | Active non-digital exercise control |
| C3 | Face-to-face professionally supervised exercise | Conventional professional supervision |
| D1 | Self-directed digital exercise without ongoing individualised human feedback | Self-directed/automated digital support |
| D2 | Digitally delivered exercise with asynchronous professional support | Non-real-time professional support |
| D3 | Digitally delivered exercise with synchronous or hybrid professional support | Real-time or hybrid professional support |

## Operational node-assignment algorithm

1. Determine whether a study-prescribed structured exercise programme was present.

2. Determine whether exercise was delivered digitally or non-digitally.

3. Determine whether planned ongoing individualised human clinical support was absent or present.

4. If human support was present, classify it as asynchronous (D2) or synchronous/hybrid (D3).

- Automated reminders, self-monitoring, algorithmic progression, and sensor-generated feedback without planned ongoing human review remained D1. One-off orientation, technical support, or generic education common to both arms did not determine node membership. Education, pain management, goal setting, and behavioural support were extracted as co-interventions and considered in the transitivity assessment; they did not override node assignment unless they altered the exercise-delivery, feedback, or professional-contact mechanism.

*Classification uncertainties examined post hoc: Kotnik 2026 digital arm D2 -> D3 because optional video consultation could plausibly be considered synchronous support; Nambi 2026 control C2 -> C3 because the report contained conflicting statements regarding weekly supervision. These alternative classifications were sensitivity analyses and did not replace the prespecified primary coding.*

| Trial_ID | Study_ID | Study | Arm | Node | Pain | Function | Retention | Status |
|---|---|---|---|---|---|---|---|---|
| T01 | 1 | Aily 2023 | A | D2 | No | Yes | Yes | Included |
| T01 | 1 | Aily 2023 | B | C3 | No | Yes | Yes | Included |
| T02 | 2 | Alasfour 2022 | A | D2 | No | Yes | Yes | Included |
| T02 | 2 | Alasfour 2022 | B | C2 | No | Yes | Yes | Included |
| T03 | 3 | Allen 2018 | A | D1 | Yes | Yes | Yes | Included |
| T03 | 3 | Allen 2018 | B | C3 | Yes | Yes | Yes | Included |
| T03 | 3 | Allen 2018 | C | C0 | Yes | Yes | Yes | Included |
| T04 | 6 | Barton 2026 | A | D3 | No | No | Yes | Included |
| T04 | 6 | Barton 2026 | B | C3 | No | No | Yes | Included |
| T05 | 7 | Bennell 2022 | A | D1 | Yes | Yes | Yes | Included |
| T05 | 7 | Bennell 2022 | B | C1 | Yes | Yes | Yes | Included |
| T06 | 8 | Bennell 2020 | A | D1 | No | No | Yes | Included |
| T06 | 8 | Bennell 2020 | B | C2 | No | No | Yes | Included |
| T07 | 9 | Cai 2026 | A | D3 | No | No | Yes | Included |

| Trial_ID | Study_ID | Study | Arm | Node | Pain | Function | Retention | Status |
|---|---|---|---|---|---|---|---|---|
| T07 | 9 | Cai 2026 | B | C3 | No | No | Yes | Included |
| T08 | 11 | Dieter 2024 | A | D1 | No | No | No | Systematic-review set only; no frozen outcome-network estimate |
| T08 | 11 | Dieter 2024 | B | Excluded: D1 + active knee brace | No | No | No | Excluded from primary node network because of additional active brace component |
| T08 | 11 | Dieter 2024 | C | C0 | No | No | No | Systematic-review set only; no frozen outcome-network estimate |
| T09 | 12 | Dieter 2025 | A | D1 | No | No | Yes | Included |
| T09 | 12 | Dieter 2025 | B | C0 | No | No | Yes | Included |
| T10 | 13 | Gohir 2021 | A | D2 | Yes | Yes | Yes | Included |
| T10 | 13 | Gohir 2021 | B | C0 | Yes | Yes | Yes | Included |
| T11 | 15 | Im 2025 | A | D2 | Yes | Yes | Yes | Included |
| T11 | 15 | Im 2025 | B | C2 | Yes | Yes | Yes | Included |
| T12 | 16 | Kotnik 2026 | A | D2 | Yes | Yes | Yes | Included; alternative D3 classification examined post hoc |
| T12 | 16 | Kotnik 2026 | B | C2 | Yes | Yes | Yes | Included |
| T13 | 19 | Lo 2024 | A | D1 | Yes | Yes | Yes | Included |
| T13 | 19 | Lo 2024 | B | C2 | Yes | Yes | Yes | Included |
| T14 | 21 | Nambi 2026 | A | D3 | No | No | Yes | Included |
| T14 | 21 | Nambi 2026 | B | C2 | No | No | Yes | Included; alternative C3 classification examined post hoc |
| T15 | 22 | Nelligan 2021 | A | D1 | No | Yes | Yes | Included |
| T15 | 22 | Nelligan 2021 | B | C1 | No | Yes | Yes | Included |
| T16 | 24 | Plavoukou 2025 | A | D3 | Yes | Yes | Yes | Included |
| T16 | 24 | Plavoukou 2025 | B | C3 | Yes | Yes | Yes | Included |
| T17 | 29 | Tedeschi 2024 | A | D3 | No | No | Yes | Included |
| T17 | 29 | Tedeschi 2024 | B | C3 | No | No | Yes | Included |
| T18 | 30 | Tore 2023 | A | D3 | No | No | Yes | Included |
| T18 | 30 | Tore 2023 | B | C2 | No | No | Yes | Included |
| T19 | 31 | Tümtürk 2024 | A | D2 | Yes | Yes | Yes | Included |
| T19 | 31 | Tümtürk 2024 | B | C2 | Yes | Yes | Yes | Included |
| T20 | 34 | White 2025 | A | D3 | No | No | Yes | Included |
| T20 | 34 | White 2025 | B | C1 | No | No | Yes | Included |
| T21 | 36 | Zhu 2026 (online 2025) | A | D1 | No | Yes | Yes | Included |
| T21 | 36 | Zhu 2026 (online 2025) | B | C1 | No | Yes | Yes | Included |

# Supplementary Table S3. Complete league table for WOMAC pain

*Effect measure: Hedges g. Entries below the diagonal are network estimates for the row node versus the column node; positive values favour the row node. Rankings and league-table patterns should be interpreted alongside sparse indirect evidence and GRADE certainty.*

| Row vs column | C0 | C1 | C2 | C3 | D1 | D2 | D3 |
|---|---|---|---|---|---|---|---|
| C0 | C0 | | | | | | |

| Row vs column | C0 | C1 | C2 | C3 | D1 | D2 | D3 |
|---|---|---|---|---|---|---|---|
| C1 | 0.15 (-0.23, 0.54) | C1 | | | | | |
| C2 | 0.17 (-0.23, 0.58) | 0.02 (-0.50, 0.54) | C2 | | | | |
| C3 | 0.15 (-0.13, 0.43) | -0.00 (-0.36, 0.35) | -0.02 (-0.48, 0.44) | C3 | | | |
| D1 | 0.30 (0.03, 0.58) | 0.15 (-0.12, 0.42) | 0.13 (-0.31, 0.57) | 0.15 (-0.08, 0.38) | D1 | | |
| D2 | 0.38 (0.03, 0.73) | 0.22 (-0.27, 0.71) | 0.21 (-0.06, 0.47) | 0.23 (-0.20, 0.65) | 0.08 (-0.33, 0.49) | D2 | |
| D3 | 0.65 (-0.03, 1.33) | 0.50 (-0.21, 1.21) | 0.48 (-0.29, 1.25) | 0.50 (-0.12, 1.12) | 0.35 (-0.31, 1.01) | 0.27 (-0.48, 1.02) | D3 |

# Supplementary Table S4. Complete league table for WOMAC physical function

*Effect measure: Hedges g. Entries below the diagonal are network estimates for the row node versus the column node; positive values favour the row node. The three mechanism-matched comparisons use different comparators and do not form a linear dose-response sequence.*

| Row vs column | C0 | C1 | C2 | C3 | D1 | D2 | D3 |
|---|---|---|---|---|---|---|---|
| C0 | C0 | | | | | | |
| C1 | -0.31 (-0.62, 0.00) | C1 | | | | | |
| C2 | -0.04 (-0.40, 0.33) | 0.27 (-0.14, 0.68) | C2 | | | | |
| C3 | 0.29 (0.03, 0.54) | 0.60 (0.32, 0.87) | 0.33 (-0.03, 0.68) | C3 | | | |
| D1 | 0.16 (-0.10, 0.43) | 0.47 (0.31, 0.64) | 0.20 (-0.17, 0.58) | -0.12 (-0.35, 0.10) | D1 | | |
| D2 | 0.43 (0.14, 0.73) | 0.74 (0.38, 1.10) | 0.47 (0.22, 0.72) | 0.15 (-0.14, 0.44) | 0.27 (-0.05, 0.59) | D2 | |
| D3 | 0.77 (0.10, 1.43) | 1.08 (0.40, 1.75) | 0.81 (0.10, 1.52) | 0.48 (-0.13, 1.10) | 0.60 (-0.05, 1.26) | 0.33 (-0.34, 1.01) | D3 |

# Supplementary Table S5. Complete league table for retention at the end of the intervention

*Effect measure: risk ratio (RR). Entries below the diagonal are network estimates for the row node versus the column node; RR > 1 indicates higher retention in the row node. The table orientation has been harmonised with the manuscript so that D1 versus C1 = 0.946, D2 versus C2 = 1.003, and D3 versus C3 = 0.971. Retention reflects completion of the scheduled assessment and must not be interpreted as exercise adherence or acceptability.*

| Row vs column | C0 | C1 | C2 | C3 | D1 | D2 | D3 |
|---|---|---|---|---|---|---|---|
| C0 | C0 | | | | | | |
| C1 | 1.000 (0.926, 1.075) | C1 | | | | | |
| C2 | 1.000 (0.926, 1.087) | 1.000 (0.926, 1.075) | C2 | | | | |
| C3 | 1.031 (0.962, 1.099) | 1.031 (0.962, 1.099) | 1.031 (0.971, 1.087) | C3 | | | |
| D1 | 0.943 (0.893, 1.010) | 0.946 (0.904, 0.990) | 0.943 (0.885, 1.010) | 0.917 (0.870, 0.980) | D1 | | |
| D2 | 1.000 (0.917, 1.087) | 1.000 (0.917, 1.087) | 1.003 (0.938, 1.072) | 0.971 (0.917, 1.042) | 1.064 (0.980, 1.149) | D2 | |
| D3 | 1.000 (0.926, 1.075) | 1.000 (0.935, 1.064) | 1.000 (0.952, 1.053) | 0.971 (0.927, 1.016) | 1.053 (0.990, 1.124) | 1.000 (0.935, 1.064) | D3 |

# Supplementary Table S6. Global inconsistency and node-splitting results

| Outcome | Common heterogeneity | Global inconsistency | Interpretation |
|---|---|---|---|
| WOMAC pain | tau2 approximately 0.000 | Joint global statistic not reportable | No small node-splitting P value, but two nominal indirect paths had SE >63 and were essentially uninformative. |
| WOMAC physical function | tau2 approximately 0.000 | chi-square=0.21; df=2; P=0.901 | No clear global/local disagreement; C1-D1 indirect evidence was extremely imprecise. |
| Retention | tau2 approximately 0.00022 | chi-square=7.61; df=5; P=0.179 | Local direct-indirect inconsistency for C1-D1 and C1-D3 (both P=0.014). |

## WOMAC pain

| Comparison | Direct coef. | Direct SE | Indirect coef. | Indirect SE | Difference | Difference SE | P |
|---|---|---|---|---|---|---|---|
| C0-C3 | -0.136435 | 0.147904 | -0.519187 | 0.908093 | 0.382752 | 0.928327 | 0.680 |
| C0-D1 | -0.281952 | 0.147862 | -0.473604 | 0.440169 | 0.191651 | 0.464341 | 0.680 |
| C0-D2 | -0.411772 | 0.198026 | -0.219814 | 0.419970 | -0.191958 | 0.464316 | 0.679 |
| C1-D1 | -0.148722 | 0.137561 | -0.604185 | 63.2681 | 0.455463 | 63.2682 | 0.994 |
| C2-D1 | -0.249394 | 0.366830 | -0.057570 | 0.284797 | -0.191824 | 0.464407 | 0.680 |
| C2-D2 | -0.187345 | 0.141560 | -0.379276 | 0.442293 | 0.191931 | 0.464395 | 0.679 |
| C3-D1 | -0.145525 | 0.119230 | -0.529007 | 0.920913 | 0.383482 | 0.928586 | 0.680 |
| C3-D3 | -0.500152 | 0.313999 | 0.299107 | 63.2276 | -0.799258 | 63.2284 | 0.990 |

## WOMAC physical function

| Comparison | Direct coef. | Direct SE | Indirect coef. | Indirect SE | Difference | Difference SE | P |
|---|---|---|---|---|---|---|---|
| C0-C3 | -0.257295 | 0.147877 | -0.390929 | 0.281984 | 0.133635 | 0.318426 | 0.675 |
| C0-D1 | -0.156212 | 0.147077 | -0.217279 | 0.399562 | 0.061067 | 0.430812 | 0.887 |
| C0-D2 | -0.476725 | 0.198745 | -0.377069 | 0.226123 | -0.099656 | 0.301050 | 0.741 |
| C1-D1 | -0.473146 | 0.083671 | -0.323983 | 36.5048 | -0.149163 | 36.5049 | 0.997 |
| C2-D1 | -0.144431 | 0.365713 | -0.225305 | 0.223610 | 0.080873 | 0.428658 | 0.850 |
| C2-D2 | -0.480349 | 0.133182 | -0.399570 | 0.407419 | -0.080779 | 0.428634 | 0.851 |
| C3-D1 | 0.107996 | 0.119172 | 0.282443 | 0.402629 | -0.174447 | 0.419998 | 0.678 |

## Retention (log-RR scale)

| Comparison | Direct coef. | Direct SE | Indirect coef. | Indirect SE | Difference | Difference SE | P |
|---|---|---|---|---|---|---|---|
| C0-C3 | 0.048627 | 0.049847 | -0.002491 | 0.059623 | 0.051118 | 0.078305 | 0.514 |
| C0-D1 | -0.067197 | 0.034579 | 0.046513 | 0.094861 | -0.113709 | 0.101035 | 0.260 |
| C0-D2 | -0.007095 | 0.105463 | 0.005305 | 0.051129 | -0.012400 | 0.117203 | 0.916 |
| C1-D1 | -0.037249 | 0.023033 | -0.210202 | 0.066617 | 0.172953 | 0.070487 | 0.014 |
| C1-D3 | -0.105745 | 0.054694 | 0.067213 | 0.044465 | -0.172958 | 0.070488 | 0.014 |
| C2-D1 | -0.091859 | 0.058799 | -0.030211 | 0.047899 | -0.061649 | 0.075753 | 0.416 |
| C2-D2 | 0.022095 | 0.044574 | -0.024405 | 0.055312 | 0.046500 | 0.070990 | 0.512 |
| C2-D3 | 0.000000 | 0.034722 | -0.007239 | 0.053164 | 0.007239 | 0.063499 | 0.909 |
| C3-D1 | -0.143633 | 0.046854 | -0.029290 | 0.043644 | -0.114343 | 0.065769 | 0.082 |

| Comparison | Direct coef. | Direct SE | Indirect coef. | Indirect SE | Difference | Difference SE | P |
|---|---|---|---|---|---|---|---|
| C3-D2 | -0.041673 | 0.045922 | -0.001360 | 0.053175 | -0.040312 | 0.070259 | 0.566 |
| C3-D3 | -0.007182 | 0.025154 | -0.101632 | 0.046564 | 0.094450 | 0.052924 | 0.074 |

*Node-splitting P values were interpreted together with effect magnitude, uncertainty, network sparsity, and clinical exchangeability. P > 0.05 was not treated as proof of complete consistency.*

# Supplementary Table S7. Complete leave-one-out estimates and non-estimable comparisons

*Each key mechanism-matched network estimate was re-estimated after sequential omission of one independent trial. Non-estimable results indicate that critical direct evidence or required network connectivity was lost. The full-network estimate column uses the final Stata 18 primary estimates reported in the revised manuscript.*

| Outcome | Comparison | Omitted ID | Omitted study | Estimable | Estimate (95% CI) | tau2 | Final primary estimate |
|---|---|---|---|---|---|---|---|
| WOMAC pain | D1 vs C1 | 3 | Allen 2018 | Yes | 0.149 (-0.120 to 0.417) | 0.0 | 0.150 (-0.120 to 0.420) |
| WOMAC pain | D1 vs C1 | 7 | Bennell 2022 | No | Not estimable | — | 0.150 (-0.120 to 0.420) |
| WOMAC pain | D1 vs C1 | 13 | Gohir 2021 | Yes | 0.149 (-0.120 to 0.417) | 0.0 | 0.150 (-0.120 to 0.420) |
| WOMAC pain | D1 vs C1 | 15 | Im 2025 | Yes | 0.149 (-0.120 to 0.417) | 0.0 | 0.150 (-0.120 to 0.420) |
| WOMAC pain | D1 vs C1 | 16 | Kotnik 2026 | Yes | 0.149 (-0.120 to 0.417) | 0.0 | 0.150 (-0.120 to 0.420) |
| WOMAC pain | D1 vs C1 | 19 | Lo 2024 | Yes | 0.149 (-0.120 to 0.417) | 0.0 | 0.150 (-0.120 to 0.420) |
| WOMAC pain | D1 vs C1 | 24 | Plavoukou 2025 | Yes | 0.149 (-0.120 to 0.417) | 0.0 | 0.150 (-0.120 to 0.420) |
| WOMAC pain | D1 vs C1 | 31 | Tümtürk 2024 | Yes | 0.149 (-0.120 to 0.417) | 0.0 | 0.150 (-0.120 to 0.420) |
| WOMAC pain | D2 vs C2 | 3 | Allen 2018 | Yes | 0.189 (-0.085 to 0.463) | 0.0 | 0.210 (-0.060 to 0.470) |
| WOMAC pain | D2 vs C2 | 7 | Bennell 2022 | Yes | 0.208 (-0.053 to 0.469) | 0.0 | 0.210 (-0.060 to 0.470) |
| WOMAC pain | D2 vs C2 | 13 | Gohir 2021 | Yes | 0.189 (-0.085 to 0.463) | 0.0 | 0.210 (-0.060 to 0.470) |
| WOMAC pain | D2 vs C2 | 15 | Im 2025 | Yes | 0.190 (-0.089 to 0.468) | 0.0 | 0.210 (-0.060 to 0.470) |
| WOMAC pain | D2 vs C2 | 16 | Kotnik 2026 | Yes | 0.357 (-0.023 to 0.737) | 0.0 | 0.210 (-0.060 to 0.470) |
| WOMAC pain | D2 vs C2 | 19 | Lo 2024 | Yes | 0.189 (-0.085 to 0.463) | 0.0 | 0.210 (-0.060 to 0.470) |
| WOMAC pain | D2 vs C2 | 24 | Plavoukou 2025 | Yes | 0.208 (-0.053 to 0.469) | 0.0 | 0.210 (-0.060 to 0.470) |
| WOMAC pain | D2 vs C2 | 31 | Tümtürk 2024 | Yes | 0.157 (-0.146 to 0.459) | 0.0 | 0.210 (-0.060 to 0.470) |
| WOMAC pain | D3 vs C3 | 3 | Allen 2018 | Yes | 0.500 (-0.103 to 1.104) | 0.0 | 0.500 (-0.120 to 1.120) |
| WOMAC pain | D3 vs C3 | 7 | Bennell 2022 | Yes | 0.500 (-0.103 to 1.104) | 0.0 | 0.500 (-0.120 to 1.120) |
| WOMAC pain | D3 vs C3 | 13 | Gohir 2021 | Yes | 0.500 (-0.103 to 1.104) | 0.0 | 0.500 (-0.120 to 1.120) |
| WOMAC pain | D3 vs C3 | 15 | Im 2025 | Yes | 0.500 (-0.103 to 1.104) | 0.0 | 0.500 (-0.120 to 1.120) |
| WOMAC pain | D3 vs C3 | 16 | Kotnik 2026 | Yes | 0.500 (-0.103 to 1.104) | 0.0 | 0.500 (-0.120 to 1.120) |
| WOMAC pain | D3 vs C3 | 19 | Lo 2024 | Yes | 0.500 (-0.103 to 1.104) | 0.0 | 0.500 (-0.120 to 1.120) |
| WOMAC pain | D3 vs C3 | 24 | Plavoukou 2025 | No | Not estimable | — | 0.500 (-0.120 to 1.120) |
| WOMAC pain | D3 vs C3 | 31 | Tümtürk 2024 | Yes | 0.500 (-0.103 to 1.104) | 0.0 | 0.500 (-0.120 to 1.120) |
| WOMAC physical function | D1 vs C1 | 1 | Aily 2023 | Yes | 0.473 (0.310 to 0.637) | 0.0 | 0.470 (0.310 to 0.640) |
| WOMAC physical function | D1 vs C1 | 2 | Alasfour 2022 | Yes | 0.473 (0.310 to 0.637) | 0.0 | 0.470 (0.310 to 0.640) |
| WOMAC physical function | D1 vs C1 | 3 | Allen 2018 | Yes | 0.473 (0.310 to 0.637) | 0.0 | 0.470 (0.310 to 0.640) |
| WOMAC physical function | D1 vs C1 | 7 | Bennell 2022 | Yes | 0.487 (0.283 to 0.691) | 0.0 | 0.470 (0.310 to 0.640) |
| WOMAC physical function | D1 vs C1 | 13 | Gohir 2021 | Yes | 0.473 (0.310 to 0.637) | 0.0 | 0.470 (0.310 to 0.640) |
| WOMAC physical function | D1 vs C1 | 15 | Im 2025 | Yes | 0.473 (0.310 to 0.637) | 0.0 | 0.470 (0.310 to 0.640) |
| WOMAC physical function | D1 vs C1 | 16 | Kotnik 2026 | Yes | 0.473 (0.310 to 0.637) | 0.0 | 0.470 (0.310 to 0.640) |
| WOMAC physical function | D1 vs C1 | 19 | Lo 2024 | Yes | 0.473 (0.310 to 0.637) | 0.0 | 0.470 (0.310 to 0.640) |

| Outcome | Comparison | Omitted ID | Omitted study | Estimable | Estimate (95% CI) | tau2 | Final primary estimate |
|---|---|---|---|---|---|---|---|
| WOMAC physical function | D1 vs C1 | 22 | Nelligan 2021 | Yes | 0.459 (0.256 to 0.661) | 0.0 | 0.470 (0.310 to 0.640) |
| WOMAC physical function | D1 vs C1 | 24 | Plavoukou 2025 | Yes | 0.473 (0.310 to 0.637) | 0.0 | 0.470 (0.310 to 0.640) |
| WOMAC physical function | D1 vs C1 | 31 | Tümtürk 2024 | Yes | 0.473 (0.310 to 0.637) | 0.0 | 0.470 (0.310 to 0.640) |
| WOMAC physical function | D1 vs C1 | 36 | Zhu 2026 | Yes | 0.474 (0.280 to 0.668) | 0.0 | 0.470 (0.310 to 0.640) |
| WOMAC physical function | D2 vs C2 | 1 | Aily 2023 | Yes | 0.485 (0.238 to 0.731) | 0.0 | 0.470 (0.220 to 0.720) |
| WOMAC physical function | D2 vs C2 | 2 | Alasfour 2022 | Yes | 0.381 (0.120 to 0.641) | 0.0 | 0.470 (0.220 to 0.720) |
| WOMAC physical function | D2 vs C2 | 3 | Allen 2018 | Yes | 0.486 (0.228 to 0.743) | 0.0 | 0.470 (0.220 to 0.720) |
| WOMAC physical function | D2 vs C2 | 7 | Bennell 2022 | Yes | 0.478 (0.233 to 0.722) | 0.0 | 0.470 (0.220 to 0.720) |
| WOMAC physical function | D2 vs C2 | 13 | Gohir 2021 | Yes | 0.474 (0.228 to 0.720) | 0.0 | 0.470 (0.220 to 0.720) |
| WOMAC physical function | D2 vs C2 | 15 | Im 2025 | Yes | 0.443 (0.185 to 0.701) | 0.0 | 0.470 (0.220 to 0.720) |
| WOMAC physical function | D2 vs C2 | 16 | Kotnik 2026 | Yes | 0.668 (0.335 to 1.001) | 0.0 | 0.470 (0.220 to 0.720) |
| WOMAC physical function | D2 vs C2 | 19 | Lo 2024 | Yes | 0.486 (0.228 to 0.743) | 0.0 | 0.470 (0.220 to 0.720) |
| WOMAC physical function | D2 vs C2 | 22 | Nelligan 2021 | Yes | 0.478 (0.233 to 0.722) | 0.0 | 0.470 (0.220 to 0.720) |
| WOMAC physical function | D2 vs C2 | 24 | Plavoukou 2025 | Yes | 0.478 (0.233 to 0.722) | 0.0 | 0.470 (0.220 to 0.720) |
| WOMAC physical function | D2 vs C2 | 31 | Tümtürk 2024 | Yes | 0.487 (0.210 to 0.764) | 0.0 | 0.470 (0.220 to 0.720) |
| WOMAC physical function | D2 vs C2 | 36 | Zhu 2026 | Yes | 0.478 (0.233 to 0.722) | 0.0 | 0.470 (0.220 to 0.720) |
| WOMAC physical function | D3 vs C3 | 1 | Aily 2023 | Yes | 0.482 (-0.121 to 1.085) | 0.0 | 0.480 (-0.130 to 1.100) |
| WOMAC physical function | D3 vs C3 | 2 | Alasfour 2022 | Yes | 0.482 (-0.121 to 1.085) | 0.0 | 0.480 (-0.130 to 1.100) |
| WOMAC physical function | D3 vs C3 | 3 | Allen 2018 | Yes | 0.482 (-0.121 to 1.085) | 0.0 | 0.480 (-0.130 to 1.100) |
| WOMAC physical function | D3 vs C3 | 7 | Bennell 2022 | Yes | 0.482 (-0.121 to 1.085) | 0.0 | 0.480 (-0.130 to 1.100) |
| WOMAC physical function | D3 vs C3 | 13 | Gohir 2021 | Yes | 0.482 (-0.121 to 1.085) | 0.0 | 0.480 (-0.130 to 1.100) |
| WOMAC physical function | D3 vs C3 | 15 | Im 2025 | Yes | 0.482 (-0.121 to 1.085) | 0.0 | 0.480 (-0.130 to 1.100) |
| WOMAC physical function | D3 vs C3 | 16 | Kotnik 2026 | Yes | 0.482 (-0.121 to 1.085) | 0.0 | 0.480 (-0.130 to 1.100) |
| WOMAC physical function | D3 vs C3 | 19 | Lo 2024 | Yes | 0.482 (-0.121 to 1.085) | 0.0 | 0.480 (-0.130 to 1.100) |
| WOMAC physical function | D3 vs C3 | 22 | Nelligan 2021 | Yes | 0.482 (-0.121 to 1.085) | 0.0 | 0.480 (-0.130 to 1.100) |
| WOMAC physical function | D3 vs C3 | 24 | Plavoukou 2025 | No | Not estimable | — | 0.480 (-0.130 to 1.100) |
| WOMAC physical function | D3 vs C3 | 31 | Tümtürk 2024 | Yes | 0.482 (-0.121 to 1.085) | 0.0 | 0.480 (-0.130 to 1.100) |
| WOMAC physical function | D3 vs C3 | 36 | Zhu 2026 | Yes | 0.482 (-0.121 to 1.085) | 0.0 | 0.480 (-0.130 to 1.100) |
| Retention | D1 vs C1 | 1 | Aily 2023 | Yes | 0.946 (0.902 to 0.991) | 0.0003138786780706536 | 0.946 (0.904 to 0.990) |
| Retention | D1 vs C1 | 2 | Alasfour 2022 | Yes | 0.946 (0.904 to 0.989) | 0.00015126395447539578 | 0.946 (0.904 to 0.990) |
| Retention | D1 vs C1 | 3 | Allen 2018 | Yes | 0.951 (0.911 to 0.994) | 0.0 | 0.946 (0.904 to 0.990) |
| Retention | D1 vs C1 | 6 | Barton 2026 | Yes | 0.946 (0.902 to 0.992) | 0.0003823223773230214 | 0.946 (0.904 to 0.990) |
| Retention | D1 vs C1 | 7 | Bennell 2022 | Yes | 0.928 (0.882 to 0.976) | 0.0 | 0.946 (0.904 to 0.990) |
| Retention | D1 vs C1 | 8 | Bennell 2020 | Yes | 0.947 (0.904 to 0.993) | 0.0002796341838780988 | 0.946 (0.904 to 0.990) |
| Retention | D1 vs C1 | 9 | Cai 2026 | Yes | 0.946 (0.904 to 0.989) | 0.00018477227112879632 | 0.946 (0.904 to 0.990) |
| Retention | D1 vs C1 | 12 | Dieter 2025 | Yes | 0.945 (0.901 to 0.992) | 0.0004102041815452705 | 0.946 (0.904 to 0.990) |
| Retention | D1 vs C1 | 13 | Gohir 2021 | Yes | 0.946 (0.903 to 0.990) | 0.00023231299956138964 | 0.946 (0.904 to 0.990) |
| Retention | D1 vs C1 | 15 | Im 2025 | Yes | 0.946 (0.904 to 0.990) | 0.00021498291325541515 | 0.946 (0.904 to 0.990) |
| Retention | D1 vs C1 | 16 | Kotnik 2026 | Yes | 0.945 (0.906 to 0.987) | 0.0 | 0.946 (0.904 to 0.990) |
| Retention | D1 vs C1 | 19 | Lo 2024 | Yes | 0.946 (0.904 to 0.990) | 0.00019355304651331708 | 0.946 (0.904 to 0.990) |
| Retention | D1 vs C1 | 21 | Nambi 2026 | Yes | 0.946 (0.900 to 0.994) | 0.0005114074891356105 | 0.946 (0.904 to 0.990) |
| Retention | D1 vs C1 | 22 | Nelligan 2021 | Yes | 0.934 (0.890 to 0.982) | 0.00014410154754642993 | 0.946 (0.904 to 0.990) |

| Outcome | Comparison | Omitted ID | Omitted study | Estimable | Estimate (95% CI) | tau2 | Final primary estimate |
|---|---|---|---|---|---|---|---|
| Retention | D1 vs C1 | 24 | Plavoukou 2025 | Yes | 0.946 (0.902 to 0.993) | 0.0004058232952444765 | 0.946 (0.904 to 0.990) |
| Retention | D1 vs C1 | 29 | Tedeschi 2024 | Yes | 0.947 (0.905 to 0.991) | 0.00019978582398528412 | 0.946 (0.904 to 0.990) |
| Retention | D1 vs C1 | 30 | Tore 2023 | Yes | 0.946 (0.902 to 0.992) | 0.00038215423365932014 | 0.946 (0.904 to 0.990) |
| Retention | D1 vs C1 | 31 | Tümtürk 2024 | Yes | 0.946 (0.902 to 0.991) | 0.00032536014169726814 | 0.946 (0.904 to 0.990) |
| Retention | D1 vs C1 | 34 | White 2025 | Yes | 0.963 (0.921 to 1.008) | 0.0 | 0.946 (0.904 to 0.990) |
| Retention | D1 vs C1 | 36 | Zhu 2026 | Yes | 0.955 (0.897 to 1.017) | 0.00042992335605259374 | 0.946 (0.904 to 0.990) |
| Retention | D2 vs C2 | 1 | Aily 2023 | Yes | 1.018 (0.939 to 1.104) | 0.0003138786780706536 | 1.003 (0.938 to 1.072) |
| Retention | D2 vs C2 | 2 | Alasfour 2022 | Yes | 0.998 (0.932 to 1.069) | 0.00015126395447539578 | 1.003 (0.938 to 1.072) |
| Retention | D2 vs C2 | 3 | Allen 2018 | Yes | 0.995 (0.933 to 1.062) | 0.0 | 1.003 (0.938 to 1.072) |
| Retention | D2 vs C2 | 6 | Barton 2026 | Yes | 1.004 (0.938 to 1.075) | 0.0003823223773230214 | 1.003 (0.938 to 1.072) |
| Retention | D2 vs C2 | 7 | Bennell 2022 | Yes | 1.002 (0.939 to 1.068) | 0.0 | 1.003 (0.938 to 1.072) |
| Retention | D2 vs C2 | 8 | Bennell 2020 | Yes | 1.008 (0.942 to 1.080) | 0.0002796341838780988 | 1.003 (0.938 to 1.072) |
| Retention | D2 vs C2 | 9 | Cai 2026 | Yes | 1.003 (0.939 to 1.072) | 0.00018477227112879632 | 1.003 (0.938 to 1.072) |
| Retention | D2 vs C2 | 12 | Dieter 2025 | Yes | 1.006 (0.939 to 1.077) | 0.0004102041815452705 | 1.003 (0.938 to 1.072) |
| Retention | D2 vs C2 | 13 | Gohir 2021 | Yes | 1.004 (0.938 to 1.075) | 0.00023231299956138964 | 1.003 (0.938 to 1.072) |
| Retention | D2 vs C2 | 15 | Im 2025 | Yes | 1.014 (0.947 to 1.087) | 0.00021498291325541515 | 1.003 (0.938 to 1.072) |
| Retention | D2 vs C2 | 16 | Kotnik 2026 | Yes | 0.969 (0.904 to 1.039) | 0.0 | 1.003 (0.938 to 1.072) |
| Retention | D2 vs C2 | 19 | Lo 2024 | Yes | 1.004 (0.939 to 1.072) | 0.00019355304651331708 | 1.003 (0.938 to 1.072) |
| Retention | D2 vs C2 | 21 | Nambi 2026 | Yes | 1.004 (0.934 to 1.080) | 0.0005114074891356105 | 1.003 (0.938 to 1.072) |
| Retention | D2 vs C2 | 22 | Nelligan 2021 | Yes | 1.003 (0.939 to 1.071) | 0.00014410154754642993 | 1.003 (0.938 to 1.072) |
| Retention | D2 vs C2 | 24 | Plavoukou 2025 | Yes | 1.007 (0.940 to 1.079) | 0.0004058232952444765 | 1.003 (0.938 to 1.072) |
| Retention | D2 vs C2 | 29 | Tedeschi 2024 | Yes | 1.009 (0.943 to 1.079) | 0.00019978582398528412 | 1.003 (0.938 to 1.072) |
| Retention | D2 vs C2 | 30 | Tore 2023 | Yes | 1.004 (0.937 to 1.075) | 0.00038215423365932014 | 1.003 (0.938 to 1.072) |
| Retention | D2 vs C2 | 31 | Tümtürk 2024 | Yes | 1.019 (0.941 to 1.103) | 0.00032536014169726814 | 1.003 (0.938 to 1.072) |
| Retention | D2 vs C2 | 34 | White 2025 | Yes | 1.000 (0.937 to 1.066) | 0.0 | 1.003 (0.938 to 1.072) |
| Retention | D2 vs C2 | 36 | Zhu 2026 | Yes | 1.004 (0.938 to 1.075) | 0.00042992335605259374 | 1.003 (0.938 to 1.072) |
| Retention | D3 vs C3 | 1 | Aily 2023 | Yes | 0.977 (0.931 to 1.025) | 0.0003138786780706536 | 0.971 (0.927 to 1.016) |
| Retention | D3 vs C3 | 2 | Alasfour 2022 | Yes | 0.974 (0.932 to 1.019) | 0.00015126395447539578 | 0.971 (0.927 to 1.016) |
| Retention | D3 vs C3 | 3 | Allen 2018 | Yes | 0.985 (0.942 to 1.031) | 0.0 | 0.971 (0.927 to 1.016) |
| Retention | D3 vs C3 | 6 | Barton 2026 | Yes | 0.972 (0.924 to 1.023) | 0.0003823223773230214 | 0.971 (0.927 to 1.016) |
| Retention | D3 vs C3 | 7 | Bennell 2022 | Yes | 0.976 (0.935 to 1.018) | 0.0 | 0.971 (0.927 to 1.016) |
| Retention | D3 vs C3 | 8 | Bennell 2020 | Yes | 0.971 (0.927 to 1.017) | 0.0002796341838780988 | 0.971 (0.927 to 1.016) |
| Retention | D3 vs C3 | 9 | Cai 2026 | Yes | 0.973 (0.930 to 1.019) | 0.00018477227112879632 | 0.971 (0.927 to 1.016) |
| Retention | D3 vs C3 | 12 | Dieter 2025 | Yes | 0.972 (0.927 to 1.019) | 0.0004102041815452705 | 0.971 (0.927 to 1.016) |
| Retention | D3 vs C3 | 13 | Gohir 2021 | Yes | 0.973 (0.930 to 1.018) | 0.00023231299956138964 | 0.971 (0.927 to 1.016) |
| Retention | D3 vs C3 | 15 | Im 2025 | Yes | 0.972 (0.929 to 1.016) | 0.00021498291325541515 | 0.971 (0.927 to 1.016) |
| Retention | D3 vs C3 | 16 | Kotnik 2026 | Yes | 0.980 (0.939 to 1.023) | 0.0 | 0.971 (0.927 to 1.016) |
| Retention | D3 vs C3 | 19 | Lo 2024 | Yes | 0.973 (0.931 to 1.018) | 0.00019355304651331708 | 0.971 (0.927 to 1.016) |
| Retention | D3 vs C3 | 21 | Nambi 2026 | Yes | 0.972 (0.925 to 1.022) | 0.0005114074891356105 | 0.971 (0.927 to 1.016) |
| Retention | D3 vs C3 | 22 | Nelligan 2021 | Yes | 0.975 (0.932 to 1.019) | 0.00014410154754642993 | 0.971 (0.927 to 1.016) |
| Retention | D3 vs C3 | 24 | Plavoukou 2025 | Yes | 0.965 (0.914 to 1.018) | 0.0004058232952444765 | 0.971 (0.927 to 1.016) |
| Retention | D3 vs C3 | 29 | Tedeschi 2024 | Yes | 0.958 (0.906 to 1.013) | 0.00019978582398528412 | 0.971 (0.927 to 1.016) |

| Outcome | Comparison | Omitted ID | Omitted study | Estimable | Estimate (95% CI) | tau2 | Final primary estimate |
|---|---|---|---|---|---|---|---|
| Retention | D3 vs C3 | 30 | Tore 2023 | Yes | 0.973 (0.928 to 1.020) | 0.00038215423365932014 | 0.971 (0.927 to 1.016) |
| Retention | D3 vs C3 | 31 | Tümtürk 2024 | Yes | 0.970 (0.926 to 1.017) | 0.00032536014169726814 | 0.971 (0.927 to 1.016) |
| Retention | D3 vs C3 | 34 | White 2025 | Yes | 0.987 (0.945 to 1.032) | 0.0 | 0.971 (0.927 to 1.016) |
| Retention | D3 vs C3 | 36 | Zhu 2026 | Yes | 0.972 (0.927 to 1.019) | 0.00042992335605259374 | 0.971 (0.927 to 1.016) |

# Supplementary Table S8. Detailed GRADE domain judgements and complete evidence profiles

*Evidence from randomised controlled trials started at high certainty and was downgraded for study limitations, inconsistency, indirectness, imprecision, and publication bias. The unit of assessment was the outcome-specific network comparison. Direct-evidence counts below refer to trials and participants contributing the direct contrast, not the size of the entire outcome network. The conventional |g| approximately 0.20 benchmark was not treated as a WOMAC-specific MCID or as the sole criterion for imprecision. Retention boundaries of 0.83 and 1.20 were contextual only and were not interpreted as adherence thresholds. Two reviewers independently re-evaluated GRADE during revision; ratings were unchanged.*

| Outcome | Comparison | Network estimate | Direct evidence | Indirect evidence | Risk of bias | Inconsistency | Indirectness | Imprecision | Publication bias | Certainty | Main judgement |
|---|---|---|---|---|---|---|---|---|---|---|---|
| WOMAC pain | C1 vs C0 | g=0.15 (-0.23 to 0.54) | 0 RCTs; indirect-only | Indirect network estimate | Serious | Not serious | Not serious | Very serious | Not formally assessed (network k=8) | Very low | Wide CI includes the null and allows effects in either direction; no direct trial informed this contrast. |
| WOMAC pain | C2 vs C0 | g=0.17 (-0.23 to 0.58) | 0 RCTs; indirect-only | Indirect network estimate | Serious | Not serious | Not serious | Very serious | Not formally assessed (network k=8) | Very low | Wide indirect-only CI includes the null and substantial uncertainty in both direction and magnitude. |
| WOMAC pain | C3 vs C0 | g=0.15 (-0.13 to 0.43) | 1 RCT; 208 participants | Additional indirect evidence available | Serious | Not serious | Not serious | Serious | Not formally assessed (network k=8) | Low | CI includes the null; certainty reduced for study limitations and imprecision. |
| WOMAC pain | D1 vs C0 | g=0.30 (0.03 to 0.58) | 1 RCT; 210 participants | Additional indirect evidence available | Serious | Not serious | Not serious | Serious | Not formally assessed (network k=8) | Low | Direction favours D1, but study limitations and uncertainty in magnitude remain. |
| WOMAC pain | D2 vs C0 | g=0.38 (0.03 to 0.73) | 1 RCT; 105 participants | Additional indirect evidence available | Serious | Not serious | Not serious | Serious | Not formally assessed (network k=8) | Low | Direction favours D2, but study limitations and a wide CI reduce certainty. |
| WOMAC pain | D3 vs C0 | g=0.65 (-0.03 to 1.33) | 0 RCTs; indirect-only | Sparse indirect estimate through D3-C3 edge | Serious | Not serious; sparse network | Not serious | Serious | Not formally assessed (network k=8) | Low | Large point estimate but very wide CI including the null; D3 is sparsely represented. |
| WOMAC pain | D1 vs C1 | g=0.15 (-0.12 to 0.42) | 1 RCT; 212 participants | Nominal indirect evidence essentially uninformative (SE≈63.27) | Serious | Not serious | Not serious | Serious | Not formally assessed (network k=8) | Low | CI includes the null; indirect evidence adds little information. |
| WOMAC pain | D2 vs C2 | g=0.21 (-0.06 to 0.47) | 3 RCTs; 204 outcome participants | Indirect evidence available (SE≈0.44) | Serious | Not serious | Not serious | Serious | Not formally assessed (network k=8) | Low | CI includes the null; direct evidence is replicated but imprecision remains. |
| WOMAC pain | D3 vs C3 | g=0.50 (-0.12 to 1.12) | 1 RCT; 42 participants | Nominal indirect evidence essentially uninformative (SE≈63.23) | Serious | Not serious; sparse network | Not serious | Serious | Not formally assessed (network k=8) | Low | Single small direct trial and wide CI including the null; indirect evidence is uninformative. |
| WOMAC physical function | C1 vs C0 | g=-0.31 (-0.62 to 0.00) | 0 RCTs; indirect-only | Indirect network estimate | Serious | Not serious | Not serious | Serious | No clear exploratory signal (P=0.223) | Low | Indirect-only estimate is imprecise and reaches the null; clinical interpretation remains uncertain. |
| WOMAC physical function | C2 vs C0 | g=-0.04 (-0.40 to 0.33) | 0 RCTs; indirect-only | Indirect network estimate | Serious | Not serious | Not serious | Very serious | No clear exploratory signal (P=0.223) | Very low | Wide indirect-only CI spans effects in both directions. |
| WOMAC physical function | C3 vs C0 | g=0.29 (0.03 to 0.54) | 1 RCT; 208 participants | Additional indirect evidence available | Serious | Not serious | Not serious | Serious | No clear exploratory signal (P=0.223) | Low | CI excludes the null but study limitations and uncertainty in magnitude remain. |
| WOMAC physical function | D1 vs C0 | g=0.16 (-0.10 to 0.43) | 1 RCT; 210 participants | Additional indirect evidence available | Serious | Not serious | Not serious | Serious | No clear exploratory signal (P=0.223) | Low | CI includes the null; certainty reduced for study limitations and imprecision. |
| WOMAC physical function | D2 vs C0 | g=0.43 (0.14 to 0.73) | 1 RCT; 105 participants | Additional indirect evidence available | Serious | Not serious | Not serious | Serious | No clear exploratory signal (P=0.223) | Low | Direction favours D2; magnitude remains uncertain and no WOMAC-specific MCID is imposed. |
| WOMAC physical function | D3 vs C0 | g=0.77 (0.10 to 1.43) | 0 RCTs; indirect-only | Sparse indirect estimate through D3-C3 edge | Serious | Not serious; sparse network | Not serious | Serious | No clear exploratory signal (P=0.223) | Low | Large point estimate but wide CI; D3 is supported by one small direct edge in this network. |
| WOMAC physical function | D1 vs C1 | g=0.47 (0.31 to 0.64) | 3 RCTs; 588 outcome participants | Indirect evidence present but extremely imprecise (SE≈36.50) | Serious | Not serious | Not serious | Not serious | No clear exploratory signal (P=0.223) | Moderate | CI excludes the null and is comparatively precise; rating remains limited by study-level risk of bias. No WOMAC-specific MCID is inferred. |

| Outcome | Comparison | Network estimate | Direct evidence | Indirect evidence | Risk of bias | Inconsistency | Indirectness | Imprecision | Publication bias | Certainty | Main judgement |
|---|---|---|---|---|---|---|---|---|---|---|---|
| WOMAC physical function | D2 vs C2 | g=0.47 (0.22 to 0.72) | 4 RCTs; 239 outcome participants | Indirect evidence available (SE≈0.41) | Serious | Not serious | Not serious | Not serious | No clear exploratory signal (P=0.223) | Moderate | CI excludes the null and direct evidence is replicated; study limitations remain the principal downgrade. |
| WOMAC physical function | D3 vs C3 | g=0.48 (-0.13 to 1.10) | 1 RCT; 42 participants | No independent informative indirect estimate (D3 leaf node) | Serious | Not serious; sparse network | Not serious | Serious | No clear exploratory signal (P=0.223) | Low | Single small direct trial and wide CI including the null. |
| Retention | C1 vs C0 | RR=1.001 (0.926 to 1.082) | 0 RCTs; indirect-only | Indirect network estimate | Serious | Not serious | Not serious | Not serious | No clear exploratory signal (P=0.874) | Moderate | Estimate is near the null; contextual 0.83-1.20 boundaries are not adherence thresholds. |
| Retention | C2 vs C0 | RR=1.000 (0.919 to 1.087) | 0 RCTs; indirect-only | Indirect network estimate | Serious | Not serious | Not serious | Not serious | No clear exploratory signal (P=0.874) | Moderate | Estimate is near the null; retention reflects study completion only. |
| Retention | C3 vs C0 | RR=1.028 (0.956 to 1.106) | 1 RCT; 208 participants | Additional indirect evidence available | Serious | Not serious | Not serious | Not serious | No clear exploratory signal (P=0.874) | Moderate | No large difference in trial completion is suggested; study limitations remain. |
| Retention | D1 vs C0 | RR=0.947 (0.886 to 1.012) | 2 RCTs; 405 participants | Additional indirect evidence available | Serious | Not serious | Not serious | Not serious | No clear exploratory signal (P=0.874) | Moderate | CI includes 1; no large difference in trial completion is suggested. |
| Retention | D2 vs C0 | RR=1.003 (0.918 to 1.096) | 1 RCT; 146 participants | Additional indirect evidence available | Serious | Not serious | Not serious | Not serious | No clear exploratory signal (P=0.874) | Moderate | Estimate is close to the null; interpretation is limited to assessment completion. |
| Retention | D3 vs C0 | RR=0.998 (0.923 to 1.080) | 0 RCTs; indirect-only | Indirect network estimate | Serious | Not serious | Not serious | Not serious | No clear exploratory signal (P=0.874) | Moderate | Indirect estimate is close to the null; does not measure exercise adherence or acceptability. |
| Retention | D1 vs C1 | RR=0.946 (0.904 to 0.990) | 3 RCTs; 596 participants | Indirect evidence available; local inconsistency P=0.014 | Serious | Serious | Not serious | Not serious | No clear exploratory signal (P=0.874) | Low | Local direct-indirect inconsistency lowers certainty; magnitude remains small and reflects study completion. |
| Retention | D2 vs C2 | RR=1.003 (0.938 to 1.072) | 4 RCTs; 277 participants | Indirect evidence available; no clear local disagreement | Serious | Not serious | Not serious | Not serious | No clear exploratory signal (P=0.874) | Moderate | Estimate is close to 1; no large difference in study completion is suggested. |
| Retention | D3 vs C3 | RR=0.971 (0.927 to 1.016) | 4 RCTs; 176 participants | Indirect evidence available; node-splitting P=0.074 | Serious | Not serious | Not serious | Not serious | No clear exploratory signal (P=0.874) | Moderate | CI includes 1 and indicates little difference in study completion; not an adherence outcome. |

# Supplementary Table S9. Arm-level intervention components and node-defining features

*This table was added to make node membership and clinical heterogeneity transparent. Each randomised arm is described separately, including exercise content, dose/progression, automated features, professional contact, education/behavioural support, and important adjuncts. The Dieter 2024 app-plus-brace arm is shown for completeness but was excluded from the primary node network because of the additional active brace component.*

| Study | Arm/node | Exercise content | Dose/progression | Digital/automated features | Human professional contact | Education/behavioural support | Other adjuncts/status |
|---|---|---|---|---|---|---|---|
| Aily 2023 [51] | D2 | Circuit training | 25-45 min, 3 sessions/week, 14 weeks; progressive circuit | Pre-recorded videos via DVD/web/YouTube/WhatsApp | PT telephone support at weeks 2, 3, 4, 6, 8, 11, and 14 | Exercise guidance | Digital delivery plus scheduled non-real-time professional support |
| Aily 2023 [51] | C3 | Same circuit training | 25-45 min, 3 sessions/week, 14 weeks | None | Face-to-face PT-supervised small-group sessions | Exercise guidance | Same exercise content; professional supervision delivered in person |
| Alasfour 2022 [61] | D2 | Home strengthening | Daily, 6 weeks; 2 exercises in week 1 progressing to 9 by weeks 5-6; 1 set of 10 repetitions | App animation, audio prompts, reminders, repetition setting and adherence logging | Therapist remotely set/monitored the programme | Behavioural prompts/reminders | Digital exercise with remote monitoring |
| Alasfour 2022 [61] | C2 | Same home strengthening | Daily, 6 weeks; same progression | Paper instructions | No ongoing professional supervision | None beyond written guidance | Non-digital self-directed comparator |
| Allen 2018 [62] | D1 | Strengthening, stretching and aerobic exercise | Strengthening/stretching >=3 days/week; aerobic activity encouraged daily; 4 months | IBET web platform, automated tailoring, video demonstrations, progression suggestions, reminders and progress graphs | No ongoing individualised human feedback | Automated self-management support | Self-directed digital programme |
| Allen 2018 [62] | C3 | Individualised PT exercise | Up to 8 PT visits (~1 h each) within 4 months | None | Face-to-face individual PT | Individualised exercise counselling | Professionally supervised comparator |
| Allen 2018 [62] | C0 | No study-prescribed structured exercise | 4-month comparison period | None | Usual clinical care only | None study-prescribed | Waiting-list/usual-care comparator |
| Barton 2026 [63] | D3 | GLA:D group exercise plus education | 1 individual assessment, 2x60-min education sessions, 12x60-min exercise sessions over 6-8 weeks | Videoconferencing (eHAB/Zoom) | Real-time group supervision, correction and individualised progression by PT | OA education/self-management | First formal follow-up at 3 months from intervention commencement |
| Barton 2026 [63] | C3 | Same GLA:D group exercise plus education | Same dose over 6-8 weeks | None | Face-to-face group PT supervision | Same OA education/self-management | Mode of professional delivery differed; content was matched |
| Bennell 2022 [64] | D1 | Online yoga | 30 min, 3 sessions/week, 12 weeks; 12 progressively released sessions | Pre-recorded videos plus automated weekly reminder/motivational emails | No ongoing individualised human feedback | Automated encouragement | Unsupervised online exercise |
| Bennell 2022 [64] | C1 | No structured exercise prescription | 12 weeks | Online knee OA education website | No ongoing professional supervision | Education on OA, exercise, weight, pain and sleep | Digital education control |
| Bennell 2020 [65] | D1 | Home exercise continued after | 3 sessions/week, 24 weeks | Automated tailored behaviour-change text | No ongoing clinician review | Barrier-targeted messages, positive | Same exercise as C2 plus automated digital |

| Study | Arm/node | Exercise content | Dose/progression | Digital/automated features | Human professional contact | Education/behavioural support | Other adjuncts/status |
|---|---|---|---|---|---|---|---|
| | | prior supervised phase | | messages, up to 5/week then tapered | | reinforcement, motivation | behaviour support |
| Bennell 2020 [65] | C2 | Same unsupervised home exercise | 3 sessions/week, 24 weeks | None; paper exercise instructions and optional log | No ongoing supervision | Written exercise guidance | Non-digital self-directed comparator |
| Cai 2026 [66] | D3 | Home exercise with wearable support | 3 sessions/week, 12 weeks | Rebee wearable sensors and app, exercise videos, completion/repetition/ROM tracking | Live video guidance at weeks 4 and 8 plus planned in-person reviews at baseline and week 12 | Exercise education | Hybrid digital-professional intervention |
| Cai 2026 [66] | C3 | Comparable home exercise | 3 sessions/week, 12 weeks | Paper exercise sheet/log | Face-to-face PT at baseline, weeks 4, 8 and 12 | Exercise education | Conventional professionally supervised pathway |
| Dieter 2024 [45] | D1 | Sensor-guided knee exercise | 25-30 min, 3 sessions/week, 12 weeks; 5 exercises/session | re.flex app, dual motion sensors, real-time audiovisual feedback, voice correction, reminders and progress tracking | No ongoing professional support | None/standard exercise information | App-only arm; retained for systematic review but did not contribute to frozen networks |
| Dieter 2024 [45] | Excluded arm | Same digital exercise plus corrective knee brace | Same 12-week digital exercise programme | Same re.flex automated feedback | No ongoing professional support | None/standard exercise information | Excluded from primary node network because the additional active brace prevented clean node assignment |
| Dieter 2024 [45] | C0 | No study-prescribed structured exercise | 12 weeks | None | Usual care | None study-prescribed | Waiting-list/usual-care comparator |
| Dieter 2025 [67] | D1 | Strengthening, ROM, stretching and balance exercise | 3 sessions/week, 12 weeks | re.flex app plus sensors; virtual-limb display, movement-quality scoring, voice feedback and push reminders | No ongoing professional supervision | Automated engagement support | Self-directed sensor-guided digital exercise |
| Dieter 2025 [67] | C0 | No study-prescribed structured exercise | 12 weeks | None | Usual care | None study-prescribed | Usual-care comparator |
| Gohir 2021 [68] | D2 | Daily strengthening, core stability and balance exercise | Daily, 6 weeks | Joint Academy app; adaptive progression, notifications and exercise tracking | Asynchronous therapist chat and/or telephone support | OA education and adherence support | Digital programme combined automated adaptation with non-real-time therapist input |
| Gohir 2021 [68] | C0 | Usual self-management/routine care | 6 weeks | None study-prescribed | GP/PT care as normally available | Usual NICE-based information/exercise advice as delivered in routine care | Comparator intensity could vary |
| Im 2025 [69] | D2 | Stretching, strengthening, functional exercise and cool-down | 30 min daily, 6 weeks | App-based delivery; daily pain/RPE reporting and weekly KOOS-PS entry | Therapist/rehabilitation physician reviewed data and adjusted intensity weekly | Monitoring and individualised dose adjustment | Asynchronous clinician-guided digital exercise |
| Im 2025 [69] | C2 | Same home exercise | 30 min daily, 6 weeks | Paper manual | One initial individual PT instruction; no ongoing remote review | Written instructions | Non-digital self-directed exercise |
| Kotnik 2026 [38] | D2 | Strengthening, ROM and balance exercise | 3 sessions/week, 12 weeks; <=72 h between sessions | Web videos; automatic reminder after >72 h inactivity; app/email messaging | PT contact available asynchronously; optional video consultation | Adherence encouragement | Primary D2 classification; alternative D3 classification examined because synchronous video contact was available |
| Kotnik 2026 [38] | C2 | Same strengthening, ROM and balance exercise | 3 sessions/week, 12 weeks | Paper booklet and exercise log | One mid-programme telephone contact for questions/adherence encouragement | Minimal support | Non-digital comparator |
| Lo 2024 [36] | D1 | Lower-limb strengthening in immersive VR | 30 min/day, 5 days/week, 12 weeks | Smartphone/headset VR, virtual coach, audiovisual feedback, level unlocking and automatic exercise logging | No ongoing human feedback | Automated motivational/game features | Self-directed digital exercise |
| Lo 2024 [36] | C2 | Same lower-limb strengthening | 30 min/day, 5 days/week, 12 weeks | Paper education/exercise manual and diary | No ongoing professional supervision | Written guidance | Non-digital self-directed comparator |
| Nambi 2026 [70] | D3 | Progressive home exercise | 45-60 min, 5 sessions/week, 8 weeks (40 sessions), progressed from lower initial frequency/duration | Rehabilitation app plus videoconferencing | Real-time PT demonstration, feedback and progression | Education within app | Synchronous digital supervision |
| Nambi 2026 [70] | C2 (primary) | Same exercise programme via paper booklet | 8 weeks | Paper booklet | Report contains conflicting statements about weekly supervision; primary coding treated it as self-directed | Written education | Alternative C3 coding examined post hoc because of the supervision ambiguity |
| Nelligan 2021 [71] | D1 | Web-based strengthening plus physical-activity programme | Strengthening 3 sessions/week for 24 weeks; increased daily physical activity encouraged | Web programme plus automated behaviour-change text messages | No ongoing individualised human feedback | Automated reminders/barrier management | Self-directed digital intervention |
| Nelligan 2021 [71] | C1 | No structured strengthening prescription | 24 weeks | Web-based OA/exercise/physical-activity education | No ongoing professional supervision | Digital education only | Digital-attention comparator |
| Plavoukou 2025 [40] | D3 | Progressive strengthening, balance and neuromuscular exercise | Supervised 2x/week (45 min) plus home exercise 3x/week for 8 weeks | KneE-PAD EMG/inertial sensors with audiovisual feedback and live movement data | Real-time PT video supervision; progression informed by weekly sensor data | Exercise guidance | Sensor-augmented synchronous telerehabilitation |
| Plavoukou 2025 [40] | C3 | Same progressive exercise | Supervised 2x/week plus home exercise 3x/week for 8 weeks | None | Face-to-face PT supervision | Exercise guidance | Content-matched conventional supervision |
| Tedeschi 2024 [43] | D3 | Therapeutic land exercise | ~20-25 min, 3 sessions/week, 4 weeks (12 sessions) | WhatsApp/FaceTime videoconferencing | Real-time PT supervision; groups <=5 | Exercise instruction | Synchronous telerehabilitation |
| Tedeschi 2024 [43] | C3 | Same therapeutic exercise | Same dose | None | Face-to-face outpatient PT supervision | Exercise instruction | Content-matched conventional rehabilitation |
| Tore 2023 [47] | D3 | Strengthening, ROM, squat, balance and stretching exercise | 45-60 min, 3 sessions/week, 8 weeks (24 sessions) | Zoom videoconferencing | Real-time PT supervision and immediate movement correction | Patient education | Synchronous digital supervision |
| Tore 2023 [47] | C2 | Same exercise programme | 45-60 min, 3 sessions/week, 8 weeks | Illustrated paper booklet | No reminders, movement correction or | Written education | Self-directed non-digital comparator |

| Study | Arm/node | Exercise content | Dose/progression | Digital/automated features | Human professional contact | Education/behavioural support | Other adjuncts/status |
|---|---|---|---|---|---|---|---|
| | | | | | ongoing supervision | | |
| Tümtürk 2024 [37] | D2 | Progressive strengthening, stretching and stabilisation | Daily programme, 8 weeks; exercises/repetitions progressed | WhatsApp exercise/education materials | Weekly telephone follow-up with symptom-based adjustment | Joint protection, pain coping and weight-control education | Asynchronous professional support |
| Tümtürk 2024 [37] | C2 | Same home exercise | Daily programme, 8 weeks | Paper booklet | No scheduled telephone follow-up | Same written education | Non-digital self-directed comparator |
| White 2025 [41] | D3 | Individualised lower-limb strengthening plus walking/step goals | 5 Zoom PT consultations over 12 weeks; strengthening 3x/week; step goal increased 10-20% as achieved | Zoom plus Fitbit step monitoring | Five individual real-time PT consultations | Pain-management education and behaviour support | Synchronous professional digital intervention |
| White 2025 [41] | C1 | Web resources without individualised PT prescription | 12 weeks | Self-directed website with exercise/physical-activity/pain-management resources | No individualised professional consultation | Education/self-management resources | Frozen C1 classification; comparator content was more intensive than simple leaflet education |
| Zhu 2026 [72] | D1 | Yang-style Tai Chi | 45 min, 3 sessions/week, 12 weeks; 12 progressively released video sessions | Online videos plus automated behaviour-change app/messages | No planned ongoing professional support | Automated barrier-management/adherence messages | Self-directed digital exercise |
| Zhu 2026 [72] | C1 | No Tai Chi prescription | 12 weeks | Same OA/exercise education website without Tai Chi videos or adherence app | No ongoing professional support | Digital education | Digital-attention comparator |

# Supplementary Table S10. Study-level outcome data used for WOMAC network meta-analysis

*All final WOMAC inputs were arm-level change scores. Negative source change values indicated improvement. The derived direct g values use the direction convention that positive g favours the first-listed node. Formulas and assumptions are provided in Supplementary Methods above; missing change-score SDs used the confirmed prespecified r = 0.50 when a study-specific correlation was unavailable.*

| Outcome | Study | Direct comparison | Arm A input | Arm B input | Derived g (95% CI) | Analysis note |
|---|---|---|---|---|---|---|
| WOMAC pain | Allen 2018 [62] | D1 versus C3 | D1: mean=-1.59, SD=3.46, n=142 | C3: mean=-1.11, SD=3.19, n=140 | 0.144 (-0.090 to 0.378) | Change-score input; shared-arm covariance handled in NMA for multi-arm trials. |
| WOMAC pain | Allen 2018 [62] | D1 versus C0 | D1: mean=-1.59, SD=3.46, n=142 | C0: mean=-0.66, SD=3.13, n=68 | 0.276 (-0.014 to 0.566) | Change-score input; shared-arm covariance handled in NMA for multi-arm trials. |
| WOMAC pain | Allen 2018 [62] | C3 versus C0 | C3: mean=-1.11, SD=3.19, n=140 | C0: mean=-0.66, SD=3.13, n=68 | 0.141 (-0.149 to 0.431) | Change-score input; shared-arm covariance handled in NMA for multi-arm trials. |
| WOMAC pain | Bennell 2022 [64] | D1 versus C1 | D1: mean=-2.50, SD=3.30, n=107 | C1: mean=-2.00, SD=3.40, n=105 | 0.149 (-0.121 to 0.418) | Change-score input; shared-arm covariance handled in NMA for multi-arm trials. |
| WOMAC pain | Gohir 2021 [68] | D2 versus C0 | D2: mean=-2.20, SD=2.30, n=48 | C0: mean=-1.20, SD=2.50, n=57 | 0.412 (0.024 to 0.800) | Change-score input; shared-arm covariance handled in NMA for multi-arm trials. |
| WOMAC pain | Im 2025 [69] | D2 versus C2 | D2: mean=-1.00, SD=3.51, n=19 | C2: mean=0.50, SD=5.61, n=10 | 0.337 (-0.434 to 1.108) | Change-score input; shared-arm covariance handled in NMA for multi-arm trials. |
| WOMAC pain | Kotnik 2026 [38] | D2 versus C2 | D2: mean=-2.63, SD=3.18, n=57 | C2: mean=-2.38, SD=3.47, n=61 | 0.075 (-0.287 to 0.436) | Change-score input; shared-arm covariance handled in NMA for multi-arm trials. |
| WOMAC pain | Lo 2024 [36] | D1 versus C2 | D1: mean=-29.37, SD=90.24, n=15 | C2: mean=-3.01, SD=113.81, n=15 | 0.250 (-0.469 to 0.968) | Change-score input; shared-arm covariance handled in NMA for multi-arm trials. |
| WOMAC pain | Plavoukou 2025 [40] | D3 versus C3 | D3: mean=-14.80, SD=7.00, n=21 | C3: mean=-11.10, SD=7.50, n=21 | 0.500 (-0.114 to 1.115) | Change-score input; shared-arm covariance handled in NMA for multi-arm trials. |
| WOMAC pain | Tümtürk 2024 [37] | D2 versus C2 | D2: mean=-19.21, SD=16.22, n=29 | C2: mean=-13.37, SD=16.14, n=28 | 0.356 (-0.168 to 0.880) | Change-score input; shared-arm covariance handled in NMA for multi-arm trials. |
| WOMAC physical function | Aily 2023 [51] | D2 versus C3 | D2: mean=-18.00, SD=10.58, n=50 | C3: mean=-17.00, SD=12.53, n=50 | 0.086 (-0.307 to 0.478) | Change-score input; shared-arm covariance handled in NMA for multi-arm trials. |
| WOMAC physical function | Alasfour 2022 [61] | D2 versus C2 | D2: mean=-5.11, SD=3.14, n=18 | C2: mean=-1.29, SD=3.10, n=17 | 1.196 (0.473 to 1.919) | Change-score input; shared-arm covariance handled in NMA for multi-arm trials. |
| WOMAC physical function | Allen 2018 [62] | D1 versus C3 | D1: mean=-3.74, SD=9.89, n=142 | C3: mean=-4.77, SD=9.35, n=140 | -0.107 (-0.340 to 0.127) | Change-score input; shared-arm covariance handled in NMA for multi-arm trials. |
| WOMAC physical function | Allen 2018 [62] | D1 versus C0 | D1: mean=-3.74, SD=9.89, n=142 | C0: mean=-2.30, SD=9.07, n=68 | 0.149 (-0.140 to 0.438) | Change-score input; shared-arm covariance handled in NMA for multi-arm trials. |
| WOMAC physical function | Allen 2018 [62] | C3 versus C0 | C3: mean=-4.77, SD=9.35, n=140 | C0: mean=-2.30, SD=9.07, n=68 | 0.266 (-0.025 to 0.557) | Change-score input; shared-arm covariance handled in NMA for multi-arm trials. |
| WOMAC physical function | Bennell 2022 [64] | D1 versus C1 | D1: mean=-9.30, SD=9.60, n=107 | C1: mean=-4.70, SD=10.80, n=105 | 0.449 (0.176 to 0.721) | Change-score input; shared-arm covariance handled in |

| Outcome | Study | Direct comparison | Arm A input | Arm B input | Derived g (95% CI) | Analysis note |
|---|---|---|---|---|---|---|
| | | | | | | NMA for multi-arm trials. |
| WOMAC physical function | Gohir 2021 [68] | D2 versus C0 | D2: mean=-7.80, SD=7.25, n=48 | C0: mean=-4.30, SD=7.32, n=57 | 0.477 (0.087 to 0.866) | Change-score input; shared-arm covariance handled in NMA for multi-arm trials. |
| WOMAC physical function | Im 2025 [69] | D2 versus C2 | D2: mean=-8.77, SD=13.18, n=19 | C2: mean=2.60, SD=15.45, n=10 | 0.791 (-0.004 to 1.585) | Change-score input; shared-arm covariance handled in NMA for multi-arm trials. |
| WOMAC physical function | Kotnik 2026 [38] | D2 versus C2 | D2: mean=-8.90, SD=12.40, n=57 | C2: mean=-5.70, SD=12.50, n=61 | 0.255 (-0.107 to 0.618) | Change-score input; shared-arm covariance handled in NMA for multi-arm trials. |
| WOMAC physical function | Lo 2024 [36] | D1 versus C2 | D1: mean=-61.50, SD=326.86, n=15 | C2: mean=-8.81, SD=380.19, n=15 | 0.145 (-0.572 to 0.861) | Change-score input; shared-arm covariance handled in NMA for multi-arm trials. |
| WOMAC physical function | Nelligan 2021 [71] | D1 versus C1 | D1: mean=-10.10, SD=10.80, n=103 | C1: mean=-4.40, SD=11.90, n=103 | 0.500 (0.222 to 0.777) | Change-score input; shared-arm covariance handled in NMA for multi-arm trials. |
| WOMAC physical function | Plavoukou 2025 [40] | D3 versus C3 | D3: mean=-49.30, SD=25.60, n=21 | C3: mean=-37.50, SD=22.30, n=21 | 0.482 (-0.132 to 1.096) | Change-score input; shared-arm covariance handled in NMA for multi-arm trials. |
| WOMAC physical function | Tümtürk 2024 [37] | D2 versus C2 | D2: mean=-16.48, SD=16.95, n=29 | C2: mean=-8.96, SD=16.27, n=28 | 0.446 (-0.080 to 0.972) | Change-score input; shared-arm covariance handled in NMA for multi-arm trials. |
| WOMAC physical function | Zhu 2026 [72] | D1 versus C1 | D1: mean=-12.00, SD=9.80, n=82 | C1: mean=-6.90, SD=11.60, n=88 | 0.471 (0.166 to 0.776) | Change-score input; shared-arm covariance handled in NMA for multi-arm trials. |

# Supplementary Table S11. Descriptive transitivity assessment across direct comparisons contributing to the WOMAC networks

*Formal covariate-balance tests and network meta-regression were not considered reliable because most direct comparisons were informed by few trials and several effect modifiers were incompletely reported. BMI and baseline symptom severity were particularly inconsistently reported, so transitivity was assessed descriptively. The table shows important observed overlap and residual imbalances rather than claiming that transitivity was empirically established.*

| Direct comparison | Outcome(s) | Contributing studies | Age | Sex | OA/radiographic severity | Duration | Exercise/co-intervention/comparator features | Potential imbalance/limitation |
|---|---|---|---|---|---|---|---|---|
| C0-C3 | Pain; function | Allen 2018 [62] | 64.3-65.7 y | Mixed sex | OA diagnosis; radiographic/clinical criteria | 4 months | D1/C3/C0 three-arm trial; C3 was individual PT; C0 waiting/usual care | Single trial; no between-study balance assessment possible |
| C0-D1 | Pain; function | Allen 2018 [62] | 64.3-65.7 y | Mixed sex | OA diagnosis; radiographic/clinical criteria | 4 months | Automated web exercise vs waiting/usual care | Single trial; comparator intensity differs substantially from active exercise nodes |
| C0-D2 | Pain; function | Gohir 2021 [68] | 65.2-68.0 y | Mixed sex | K-L >= I | 6 weeks | Adaptive app + therapist chat/telephone vs usual self-management/routine care | Routine-care comparator could include exercise/advice and varied by participant |
| C1-D1 | Pain; function | Bennell 2022 [64]; Nelligan 2021 [71]; Zhu 2026 [72] | ~59-63 y | Mixed sex | Clinical knee OA; radiographic severity variably reported | 12-24 weeks | Structured yoga/strengthening/Tai Chi vs digital OA education; automated behavioural support varied | Relatively aligned comparator type, but exercise content, duration and automated support varied |
| C2-D1 | Pain; function | Lo 2024 [36] | Median 63-64 y | Mixed/NR | ACR clinical/radiographic OA | 12 weeks | Immersive VR strengthening vs paper-based same exercise | Single small trial; digital modality and treatment expectations may differ |
| C2-D2 | Pain; function | Alasfour 2022 [61]; Im 2025 [69]; Kotnik 2026 [38]; Tümtürk 2024 [37] | ~51.5-71.4 y; Kotnik 50-79 y | One women-only trial; others mixed/NR | K-L/clinical OA severity varied | 6-12 weeks | Digital exercise with remote monitoring/contact vs paper/self-directed exercise | Largest imbalance: age/sex mix, professional-contact intensity, reminders, and behavioural support varied |
| C3-D1 | Pain; function | Allen 2018 [62] | 64.3-65.7 y | Mixed sex | OA diagnosis; radiographic/clinical criteria | 4 months | Automated self-directed web exercise vs individual face-to-face PT | Single trial; substantial difference in professional contact and treatment expectations |
| C3-D2 | Function only | Aily 2023 [51] | 53-55 y | Mixed sex | K-L II-III | 14 weeks | Same circuit training; pre-recorded remote programme + scheduled telephone support vs face-to-face supervised circuit | Exercise content matched relatively well; delivery/professional-contact mode differed |
| C3-D3 | Pain; function | Plavoukou 2025 [40] | Eligibility 40-70 y; reported age internally inconsistent | Mixed/NR | K-L I-III | 8 weeks | Sensor-augmented live telerehabilitation vs face-to-face supervision of the same progressive programme | Single 42-participant trial; age reporting inconsistency and sparse evidence |

# Supplementary Table S12. Additional structural and risk-of-bias sensitivity analyses

*The high-risk-of-bias and alternative-node sensitivity analyses were rerun in Stata 18 using the frozen dataset during revision; the values below agree with the revised manuscript. Analyses that were not applicable or were intentionally not imposed post hoc are stated explicitly.*

| Outcome | Sensitivity analysis | Key mechanism-matched estimates | Interpretation |
|---|---|---|---|
| WOMAC pain | Primary | D1 vs C1: g=0.15 (-0.12 to 0.42); D2 vs C2: g=0.21 (-0.06 to 0.47); D3 vs C3: g=0.50 (-0.12 to 1.12) | Frozen primary Stata 18 network |
| WOMAC pain | Exclude five trials at overall high risk of bias | Network fragmented; D1 vs C1 not estimable. Remaining C2-D1-D2 component: D2 vs C2 g=0.17 (-0.13 to 0.46). D3 vs C3 remained g=0.50 (-0.12 to 1.12). | Connectivity depended partly on high-risk trials |
| WOMAC pain | Alternative node: Kotnik 2026 D2 -> D3 | D1 vs C1 g=0.15 (-0.12 to 0.42); D2 vs C2 g=0.29 (-0.08 to 0.66); D3 vs C3 g=0.26 (-0.19 to 0.70) | D3-related estimate sensitive to plausible node definition |
| WOMAC physical function | Primary | D1 vs C1: g=0.47 (0.31 to 0.64); D2 vs C2: g=0.47 (0.22 to 0.72); D3 vs C3: g=0.48 (-0.13 to 1.10) | Frozen primary Stata 18 network |
| WOMAC physical function | Exclude five trials at overall high risk of bias | D1 vs C1 g=0.49 (0.28 to 0.69); D2 vs C2 g=0.44 (0.17 to 0.72); D3 vs C3 g=0.48 (-0.13 to 1.10) | C0 no longer represented; all remaining trials still had at least some concerns |
| WOMAC physical function | Alternative node: Kotnik 2026 D2 -> D3 | D1 vs C1 g=0.47 (0.31 to 0.64); D2 vs C2 g=0.56 (0.24 to 0.88); D3 vs C3 g=0.08 (-0.33 to 0.49) | D3 estimate materially node-definition sensitive |
| Retention | Primary | D1 vs C1 RR=0.946 (0.904 to 0.990); D2 vs C2 RR=1.003 (0.938 to 1.072); D3 vs C3 RR=0.971 (0.927 to 1.016) | Frozen primary Stata 18 network |
| Retention | Alternative nodes: Kotnik D2 -> D3; Nambi control C2 -> C3 | D1 vs C1 RR=0.94 (0.90 to 0.99); D2 vs C2 RR=0.98 (0.91 to 1.06); D3 vs C3 RR=0.99 (0.95 to 1.03) | All estimates remained close to 1 |
| WOMAC pain and function | Change-score vs endpoint data type | Not applicable: all frozen WOMAC arm-level inputs used in the primary networks were change-score inputs. | The prespecified endpoint fallback was not required in the final WOMAC networks |
| WOMAC pain and function | Duration/comparator-intensity cut-points | Not performed as a post hoc dichotomisation because no thresholds were prespecified and additional splitting would further fragment already sparse networks. | Duration and comparator intensity were instead examined in the transitivity assessment (S11) |

# Supplementary Table S13. Direct, indirect, and network evidence for comparisons highlighted by inconsistency diagnostics

*Direct and indirect coefficients are presented on the model scale used in node splitting (Hedges g for WOMAC; log-RR for retention). For sparse leaf-like WOMAC nodes, very large indirect SEs indicate that nominal network connectivity did not provide clinically informative independent indirect evidence. For retention, multiple overlapping loops prevented attribution of local inconsistency to a single study.*

| Outcome | Comparison | Direct evidence studies | Direct estimate | Indirect estimate | Network estimate | Node-splitting P | Interpretation/loops |
|---|---|---|---|---|---|---|---|
| WOMAC pain | C1-D1 | Bennell 2022 [64] | Direct coef -0.149 (SE 0.138) | Indirect coef -0.604 (SE 63.268) | Network D1 vs C1 g=0.15 (-0.12 to 0.42) | 0.994 | C1 was effectively leaf-like in the pain network; no stable clinically informative independent loop. The nominal indirect estimate was essentially uninformative. |
| WOMAC pain | C3-D3 | Plavoukou 2025 [40] | Direct coef -0.500 (SE 0.314) | Indirect coef 0.299 (SE 63.228) | Network D3 vs C3 g=0.50 (-0.12 to 1.12) | 0.990 | D3 was supported by a single direct pain trial; the nominal indirect pathway was essentially uninformative. |
| WOMAC physical function | C1-D1 | Bennell 2022 [64]; Nelligan 2021 [71]; Zhu 2026 [72] | Direct coef -0.473 (SE 0.084) | Indirect coef -0.324 (SE 36.505) | Network D1 vs C1 g=0.47 (0.31 to 0.64) | 0.997 | Indirect evidence was extremely imprecise and contributed little to certainty; the network estimate was driven mainly by direct evidence. |
| Retention | C1-D1 | Bennell 2022 [64]; Nelligan 2021 [71]; Zhu 2026 [72] | Direct coef -0.037 (SE 0.023) | Indirect coef -0.210 (SE 0.067) | Network D1 vs C1 RR=0.946 (0.904 to 0.990) | 0.014 | Local inconsistency. Principal short indirect loops included C1-D1-C2-D3-C1 and C1-D1-C3-D3-C1; overlapping paths prevent attribution to one trial. |
| Retention | C1-D3 | White 2025 [41] | Direct coef -0.106 (SE 0.055) | Indirect coef 0.067 (SE 0.044) | Network D3 vs C1 approximately RR=1.00 (approximately 0.93 to 1.08) | 0.014 | Local inconsistency. Principal indirect paths included C1-D1-C2-D3 and C1-D1-C3-D3, involving multiple trials; no single causal loop can be isolated. |

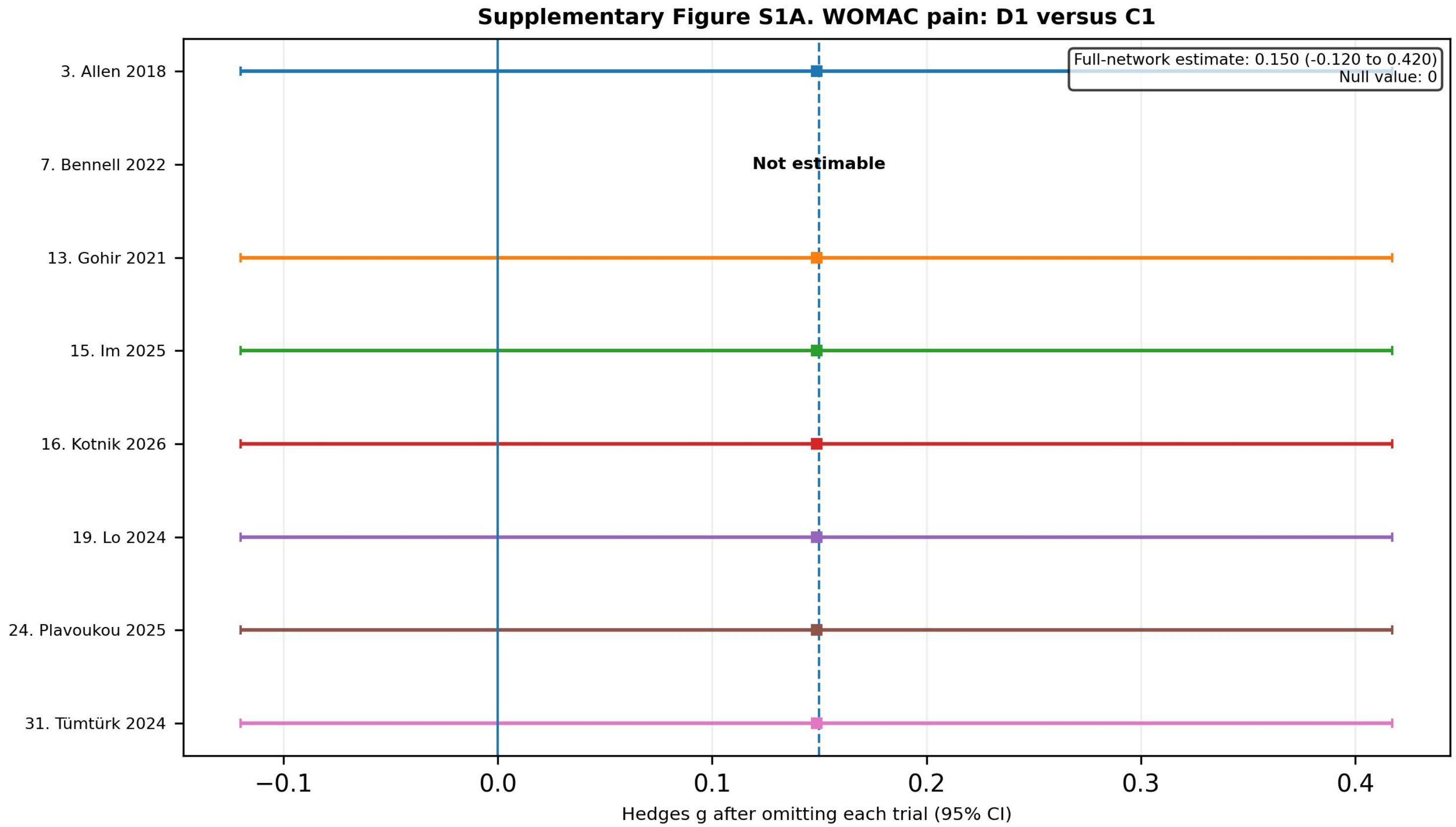


**Supplementary Figure S4A. Comparison-adjusted funnel plot for WOMAC pain. Exploratory visualisation only; k=8; no regression test. These diagnostics are exploratory and do not exclude publication bias.**

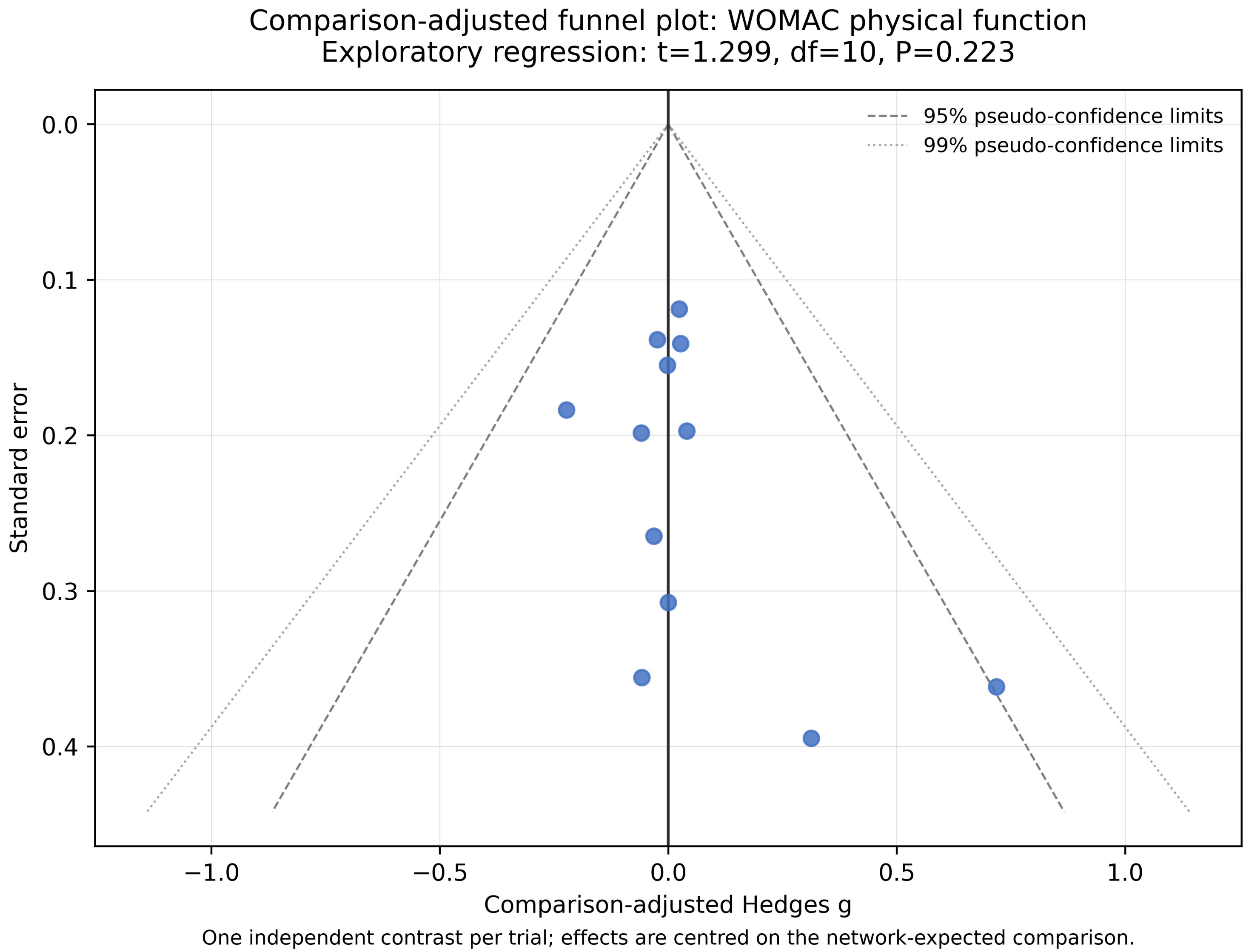


**Supplementary Figure S4B. Comparison-adjusted funnel plot for WOMAC physical function. Exploratory regression: t=1.299, df=10, P=0.223. These diagnostics are exploratory and do not exclude publication bias.**

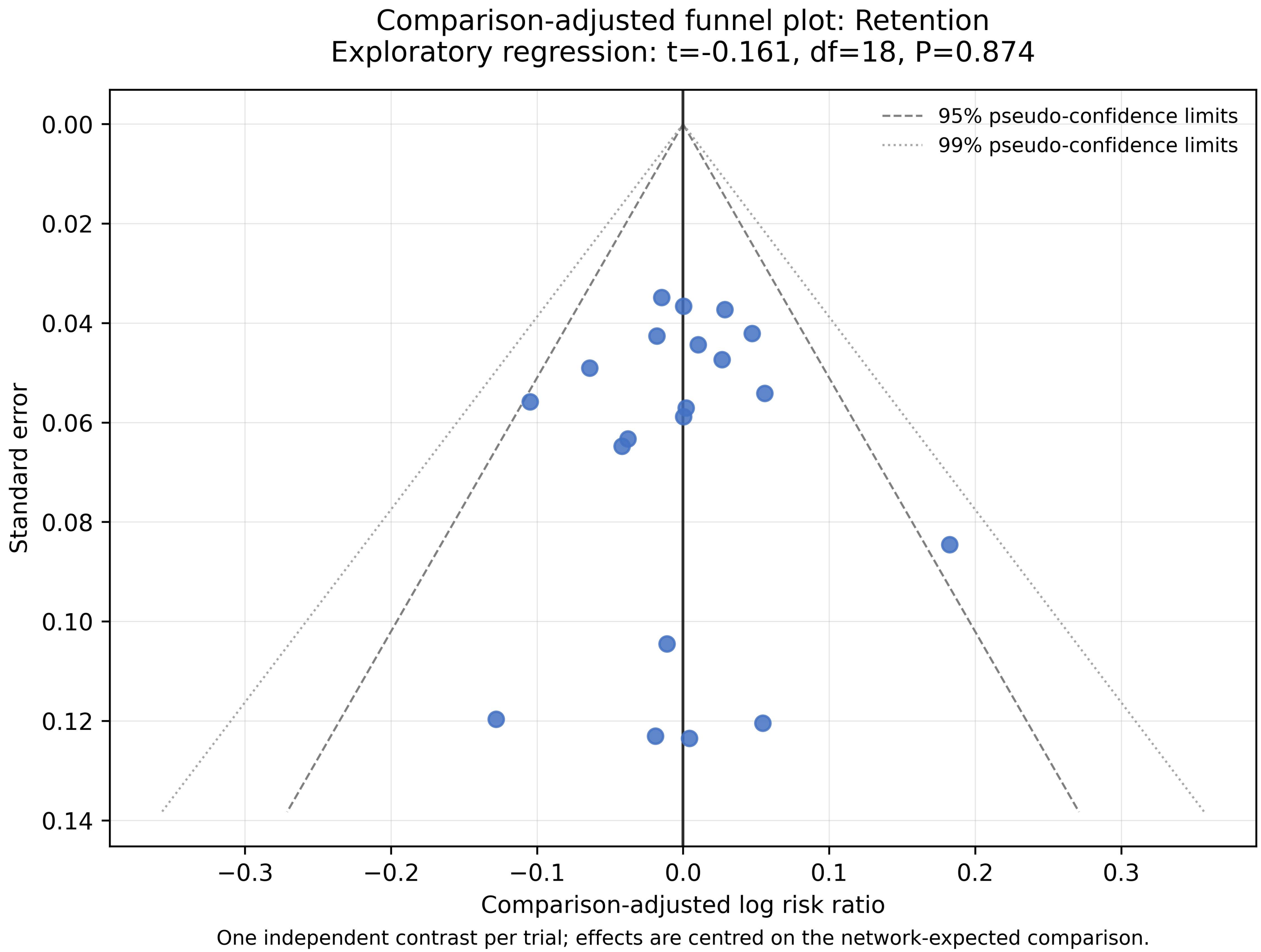


**Supplementary Figure S4C. Comparison-adjusted funnel plot for Retention. Exploratory regression: t=-0.161, df=18, P=0.874. These diagnostics are exploratory and do not exclude publication bias.**

Supplementary Materials
